# Biological principles for music and mental health

**DOI:** 10.1038/s41398-023-02671-4

**Published:** 2023-12-04

**Authors:** Daniel L. Bowling

**Affiliations:** 1 Department of Psychiatry and Behavioral Sciences, Stanford University, School of Medicine, Stanford, CA USA; 2 Center for Computer Research in Music and Acoustics (CCRMA), Stanford University, School of Humanities and Sciences, Stanford, CA USA

**Keywords:** Neuroscience, Physiology, Human behaviour, Psychiatric disorders

## Abstract

Efforts to integrate music into healthcare systems and wellness practices are accelerating but the biological foundations supporting these initiatives remain underappreciated. As a result, music-based interventions are often sidelined in medicine. Here, I bring together advances in music research from neuroscience, psychology, and psychiatry to bridge music’s specific foundations in human biology with its specific therapeutic applications. The framework I propose organizes the neurophysiological effects of music around four core elements of human musicality: tonality, rhythm, reward, and sociality. For each, I review key concepts, biological bases, and evidence of clinical benefits. Within this framework, I outline a strategy to increase music’s impact on health based on standardizing treatments and their alignment with individual differences in responsivity to these musical elements. I propose that an integrated biological understanding of human musicality—describing each element’s functional origins, development, phylogeny, and neural bases—is critical to advancing rational applications of music in mental health and wellness.

## Introduction

Every day, hundreds of millions of people make or listen to music. This appetite is driven by music’s core effects on emotion [1–3], reward [4], and affiliation [5]. The value we place on these effects supports a 200 billion dollar per year industry in the US alone [6]. More and more, music’s core effects have come into focus for their alignment with core dimensions of mental health, e.g., mood, motivation, pleasure, and social functioning. Together with rapidly increasing awareness of mental health’s humanistic and financial importance, this alignment has sparked new investments in music-based interventions from government and industry [7–9]. This interest presents an opportunity for proponents of music’s therapeutic value to increase the specificity and rigor of its application and enhance our understanding of its clinical scope and efficacy.

Meeting this goal depends on a clear conception of music’s underlying biology as a source of principles for systematic applications towards specific clinical and subclinical goals. An awareness of such principles exists in music therapy [10–12], especially “neurologic” music therapies for motor rehabilitation [13–15], but applications in mental health remain highly variable, making it difficult to achieve a unified biologically-informed approach. Moreover, there are far too few music therapists to meet current mental health needs. In the US, for example, there are only about 10,000 board-certified music therapists, compared to about 58 million adults living with mental illness [16, 17]. Assuming an average weekly caseload of 30 patients [18], total capacity to treat is therefore just 0.5%. Musicians represent another important source of insight, as they are ultimately the most skilled at titrating music’s neurophysiological impact. However, the inherently subjective nature of their “artistic” approach can preclude direct integration within a scientific model of health.

Given the uncertainty in defining the relationship between music and health, funders have sought to advance applications by casting a wide net. The National Institutes of Health, for example, has sponsored an extensive list of research topics involving music, including improving treatment response in cancer, stress and pain management in surgery, affect modulation in mood disorders, anxiolysis in anxiety disorders, social functioning in neurodevelopmental disorders, palliative care in advanced illness, neural rehabilitation after injury, and wellness through exercise [19]. This breadth is likely to puzzle many medical professionals and raise skepticism in more than a few. *Can music really be such a panacea?*

While skepticism is justified (as discussed in Section “Skepticism and need”), clear evidence of music’s effects on core mental health variables is readily apparent in our growing understanding of music’s biological foundations. Critically, these foundations provide a rational basis for standardizing and expanding music’s psychiatric applications and benefits. In this review, I outline a framework for music in human biology and describe some of its basic implications for standardized music-based interventions in mental health, with the goal of increasing biomedical integration and impact.

## Developing a biological perspective

As far as we know, music has been with humans since our earliest existence. The first known evidence of human preoccupation with music comes from Stone Age flutes, carefully carved in wing bones and mammoth ivory some 40,000 years ago [20]. Over the course of recorded history, explanations of music and its power have been sought in terms of mythology, cosmology, mathematics, and physics, with many important insights along the way [21, 22]. However, it was not until the 19th century that music came to be viewed in terms of human evolution. In 1871, based on observations of general similarity between human and animal vocalization, as well as the behavior of other “singing” mammals (like gibbons and howler monkeys), Darwin postulated a basis for music in sexual selection on social behavior. Specifically, he proposed that the vocalizations of our ancestors were likely more musical than linguistic, comprising greater regularity in pitch and time, and functioning mainly in signaling affect, attracting mates, and threatening rivals [23]. From this perspective, “music” provided the foundation for the evolution of human language, centering its underlying biology within the study of human cognition and communication more broadly [24].

Two aspects of this early account continue to shape modern biological music research or *biomusicology* (e.g. [24–41]). One is that music is, first and foremost, a form of social communication, with explicit origins in auditory-vocal interaction. The second is that singing and speaking—and thus, music and language—likely share a common origin in early hominids, as reflected by their many overlapping features, like being auditory-vocal by default, emotional expressive, and inherently social [25]. While many more specific details about the evolutionary origins of music remain under debate (cf [31, 38, 42–48]), a general view of music as rooted in social communication, with close ties to speech and language, is consistent across most theories and also central here.

Before proceeding, it is important to clarify that biomusicology chiefly concerns musicality rather than music per se. Whereas *music* is a cultural phenomenon of infinite variety [46], *musicality* is the genetically constrained and reliably developing set of neural capacities on which music production and perception rests [33]. It should be noted that this view departs significantly from common conceptions of music that center specific cultural manifestations and individual variation in preferences. Instead, a biological perspective centers music’s basic features in relation to pressures to evolve and develop neural capacities that support social communication. The following sections define this perspective with respect to four core elements of musicality—tonality, rhythm, reward, and sociality—reviewing essential concepts, biological bases, and evidence of clinical benefits, towards a framework for rational clinical translation.

## Tonality

### Musical terms and definitions

*Tones* are a special class of sound stimuli that evoke a strong sense of pitch. Physically, they comprise regularly spaced pressure waves that repeat at frequencies between approximately 30–4000 Hz [49]. All musical cultures and traditions use tones [50, 51], making neural sensitivity to *tonality—*defined simply as the use of tones to make music—a core element of human musicality. Tonality has primarily been considered from three perspectives. *Harmony* is focused on the organization of tones with respect to frequency. *Melody* is focused on the sequential organization of tones over time. *Timbre* is focused on the quality imparted to tones by their source and manner of production (e.g., a voice or a synthesizer, sounded gently or harshly, etc.) [52].

### Conserved aspects of tonality

**Fig. 1:**
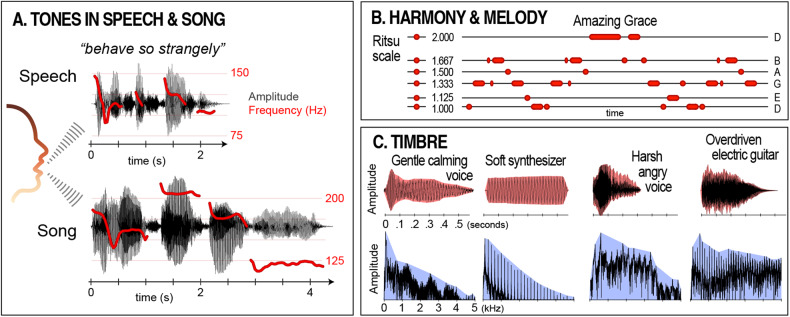
Tonality. **A** The same phrase spoken and sung by the same person to highlight how tones in music are related to tones in speech (based on Diana Deustch’s speech-to-song illusion). Variation in sound pressure over time (black) is overlaid with variation in the fundamental frequency of vocal fold vibration (the physical correlate of voice pitch; red). **B** On the left, the frequency relationships defined by the Japanese ritsu scale are presented along a vertical axis. Each relationship is calculated with respect to the lowest tone in the set (labeled “1.000”). On the right, the melody of the American gospel song “Amazing Grace” is shown using the same relationships. Conventional note letter names are listed at the right. **C** Timbral similarity of vocal and instrumental tones with parallel affective qualities. Top row: sound pressure waveforms with temporal envelopes shown in red. Bottom row: corresponding Fourier transforms with spectral envelopes shown in blue. These examples were selected to show similarity in temporal and spectral features of vocal and instrumental tones with parallel affective qualities.

The most significant source of tones in the human auditory environment is vocal fold vibration in the larynx [53, 54]. In speech, the frequency of vocal fold vibration fluctuates rapidly, leading to dynamic and variable tones (Fig. 1A). In contrast, during song, these vibrations are modulated to emphasize particular frequencies and frequency relationships [50, 51, 55]. Beyond these “universal” features, many key aspects of harmony, melody, and timbre are widely observed across richly differentiated musical cultures and traditions.

In harmony, music almost always emphasizes a small set of tones defined by specific relationships to each other [51]. The simplest of these relationships—e.g., octaves (2:1) and fifths (3:2)—feature prominently in music worldwide [21, 56, 57], and particular sets of ratios called scales (or modes) are strikingly popular across cultural boundaries [21, 57, 58]. For example, the Western minor mode corresponds to what South Indian musicians call the Keeravani raga [59]. Similarly, the Japanese ritsu scale is also found in traditional Western folk songs like “Auld Lang Syne” and “Amazing Grace” (Fig. 1B) [60]. In melody, tones tend to be arranged in arched or descending contours [21, 51], traced mainly by small steps in pitch, with larger steps typically rising (Fig. 1B) [61–64].

In timbre, specific temporal and spectral characteristics of tones give rise to specific perceptions of anatomical and affective source parameters, e.g., the ratio of low- to high-frequency energy in a tone is associated with size, valence, and arousal [65, 66], rapid tone onsets signal a higher commitment of energy [67], and “rough” growl-like tones often convey anger or aggression [68, 69] (Fig. 1C). There is also widespread conservation in the use of tones for specific purposes. For example, lullabies typically comprise tones with relatively more low-frequency energy, sorted into simple repeating patterns [70–72]. Likewise, flatter contours with narrower pitch steps are favored for conveying somber affect [63, 73]. Together, these and other broadly conserved aspects of tonality indicate a strong foundation in our shared biology.

### Biological foundations of tonality

To model the biology underlying tonality, music scientists have developed vocal similarity theory (VST), the central tenet of which is that we perceive tones according to their behavioral significance in vocal communication [22, 30, 53, 58, 74–78]. VST is based on the fact that our experience with tones is dominated by the voice at evolutionary and individual time scales. This implies that the neurobiology of tone perception has primarily been shaped by pressure to contend with tones in the voice and their significance for adaptive behavior [22, 53, 75].

Phylogenetically, sensitivity to “tone of voice” is likely to have emerged very early in tetrapod evolution [79]. In mammals, auditory-vocal interaction is often central to social behavior and cognition, placing this sensitivity under intense selective pressure. Developmentally, the fetal brain begins responding to mother’s voice around the 24th week of gestation [80]. Over the ensuing weeks, these responses develop to the point that infants strongly prefer their mother’s voice at birth [81], an orientation that scaffolds the formation of our prototypical social bond, the modulation of affect through sound, and the development of communication more broadly [82]. Mechanistically, neural specialization for responding to vocal tones is evident throughout the auditory system, from enhanced representations of periodicity in the brainstems of humans and rats [83, 84], to harmonically sensitive neurons in marmoset cortex [85], and pitch contour neurons in human cortex [86].

The culmination of this underlying biology is a brain that responds to tones reflexively by supplying percepts of meaning and intent as guides for behavior and cognition. This works because the acoustics of laryngeal vocalization are linked to source parameters at a statistical level [87, 88]. For music, the implication of VST is that conserved aspects of tonality can be understood as consequences of the auditory system’s biological tuning to voices.

### Applications of tonality in mental health

VST roots tonality in the *bioacoustics* of vocal affect, providing a principled basis for the assessment and manipulation of reflexive responses to musical tones, and their translation to psychiatry. For any given clinical goal related to the modulation of patient affect, VST predicts that proper applications of tonality require alignment with the statistical regularities that identify vocal expressions as conveying the emotion required to effect the desired physiological change. For example, a musical intervention aimed at relieving high anxiety and agitated negative mood should have tonal properties that align with a positive calming voice, such as extended falling pitch contours and low-frequency weighted timbres. Similarly, an intervention for depression should possess a gentle affirming tone, captured by more articulated contours that rise towards their ends. This approach naturally imbues musical tonality with a capacity to modulate listener feelings that parallels the corresponding tone of voice. However, because musical tones are (often) freed from the constraints of vocal expression—e.g., by instrumental production or release from linguistic demands—key regularities can be distilled and exaggerated to yield tones with supernormal neurophysiological effects.

Importantly, guidance derived from VST on how to use tonality to modulate affect largely corresponds with what musicians and music therapists have learned to do through subjective exploration and experience [76, 89]. This is reflected in the effects of current musical treatments on dysregulated anxiety and mood. For example, *receptive* treatments (based on listening) can effectively reduce acute anxiety in chemotherapy [90], childbirth [91], and surgery [92]. A 2018 meta-analysis of 81 randomized controlled studies, involving over 6000 patients, found that music listening before, during, or after surgery significantly reduced anxiety symptoms, with an effect size equal to 69% of one standard deviation (Standard Mean Difference [SMD] = 0.69) [92]. Other meta-analyses indicate that music therapy can also be an effective anxiolytic beyond these acute medical contexts. A 2021 meta-analysis of 32 controlled studies with over 1,900 patients with anxiety showed significant anxiety reduction after an average of 7.5 music therapy sessions (SMD = 0.36). This effect was stronger in the subset of 11 studies with >12 sessions (SMD = 0.59), suggesting a dose-response effect [93]. For context, consider that estimated summary SMDs for first-line psychotherapies and pharmacotherapies lie between 0.28–0.44 and 0.33–0.45 respectively (but note that these numbers are based on much larger samples) [94].

Similarly positive effects of music therapy have been reported for affect disorders. A 2017 meta-analysis of 9 controlled studies including 411 patients diagnosed with a depressive disorder found that adding 6–12 weeks of music therapy to antidepressants and/or psychotherapy significantly reduced clinician-rated and patient-rated symptoms (SMD = 0.98 and 0.85 respectively) [95]. A 2020 meta-analysis focused specifically on receptive musical treatments found an even stronger effect when looking at depressive symptoms across patients with a wider variety of primary diagnoses, like heart disease, dementia, insomnia (SMD = 1.33, 17 controlled studies, 1,284 patients) [96]. The same paper also reports a significant effect for *interactive* treatment (based on making music; SMD = 0.57, 20 controlled studies, 1,368 patients) [96]. Both effects were apparent across variable depression severity levels and treatment courses (mean dosage was approximately 14 h, SD = 18, range = 0.33–126) [95, 96]. For context, overall SMDs for psychotherapy and pharmacotherapy in depressive disorders have been estimated at 0.31 and 0.30 respectively (again, based on larger samples) [94].

While success of this kind might suggest that music therapy can do without VST, it should be noted that none of the aforementioned meta-analyses (and few of the individual studies that they cite) provide any details on the parameters of the music employed. This is largely because musical decisions are made on intuition rather than principle. Thus, while subjectivity has proven an essential guide in discovering music’s therapeutic applications, it also complicates scientific efforts to understand music’s therapeutic effects and standardize their application. VST addresses this challenge by providing objective guidelines for musical tonality based on specific therapeutic goals. This is a necessary step towards standardization, which is in turn required for expanding access to musical treatment.

## Rhythm

### Musical terms and definitions

*Rhythm* is the temporal patterning of sounds in music. The dominant feature of rhythm is temporal predictability, focused at rates ranging from approximately 0.5 to 5 Hz (30–300 beats per minute [bpm]) [97–99]. All musical cultures and traditions exhibit some temporal predictability in this range, making neural sensitivity to rhythm a second core element of musicality (no ranking implied) [50, 51]. Investigations of rhythm typically identify two core components [100]. *Pulse* is the main cycle of rhythmic repetition perceived in music; it is generally what we synchronize to when we move in time with music. *Meter* refers more broadly to other rhythmic cycles perceived in music [101]. These encompass repetition rates that are both faster and slower than the pulse, defined by subdivisions of the pulse and multi-pulse cycles, respectively.

### Conserved aspects of rhythm

**Fig. 2:**
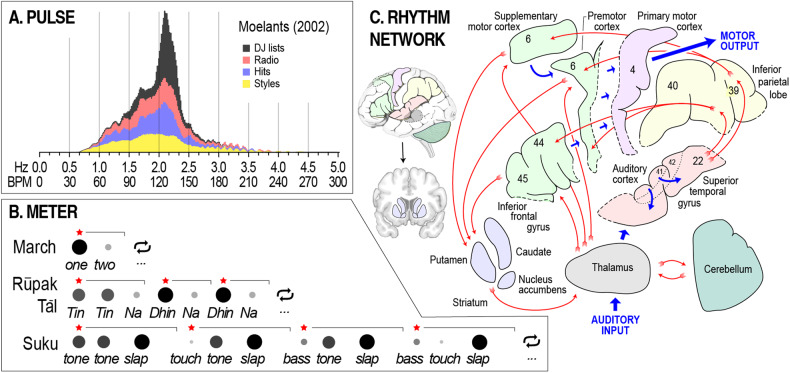
Rhythm. **A** A histogram of tempos from a sample of over 74,000 pieces of music. “DJ lists” refers to lists of song tempos used by disk jockeys to match pulse rates between tracks; “Radio” refers to songs found by randomly tuning into radio stations circa 2002; “Hits” refers to popular music from 1960–1990; and “styles” refers to a selection of music from divergent styles (e.g., renaissance polyphony and modern jazz). **B** One cycle from each of three rhythms with different meters, increasing in complexity from top to bottom. Circle size and shading indicate level of accenting (large/dark = strong), red stars and horizontal black brackets mark subgroups, and ellipsis denote repetition. *Tin, Na*, and *Dhin* are specific *tabla* drum strokes; *tone, slap, bass*, and *touch* are specific *djembe* drum stokes. The *suku* rhythm is based on section 5.3 of Polak (2010), with a timing ratio of 11:17:22 for the short-medium-long pulse patterns. **C** Hypothesized information flow through the network of brain areas implicated in rhythm perception. Additionally relevant brain areas include the hypothalamus, insula, and orbitofrontal cortex (see Fig. 3). The rhythm network is mostly bilateral despite being visualized in the left hemisphere here. Numbers refer to Brodmann areas. Insets show implicated structures in situ. Panel **A** is adapted from Moelants (2002) with permission from the author.

As with tonality, key elements of rhythm are widely conserved across musical cultures and traditions. In pulse, acceptable rates (or *tempos*) are highly constrained, showing a peak between approximately 1.33 and 2.67 Hz (80–160 bpm) across a variety of different musical traditions (Fig. 2A) [98, 102]. Intriguingly, this peak corresponds closely with dominant rates of periodicity in full-body human motion (e.g., 1.35–2.5 Hz [81–150 bpm] in walking) [98]. A second widely conserved aspect of pulse is that individual pulses tend to be *isochronous* or equally spaced in time [50, 51]. There are traditions that also use unequal pulse spacing [103], but only in ways that retain predictability and thus allow interpersonal synchrony [104, 105].

In meter, rhythmic cycles that are faster than the pulse also exhibit characteristic rates, mostly in the range of 2–8 Hz (120–480 bpm; typical of finger or wrist motion), and involving subdivisions of the pulse rate by factors of two or three [99, 101]. Faster cycle rates are found in some traditions, e.g., 10–15 Hz [600–900 bpm] in djembe [103] or death metal [106], but this is relatively rare. For cycles at rates slower than the pulse, rhythmic patterning is almost always marked by variations in acoustic emphasis called *accenting* [100] (Fig. 2B). A simple example of accenting comes from the marching rhythm “***one***, *two*, ***one***, *two*, ”, a repeating two-pulse cycle in which the first pulse is accented. Increasing in complexity, the meter of rūpak tāl in North Indian music is defined by a repeating seven-pulse cycle with multiple levels of accent set into groups of three and two [107]. More complex still are the drum patterns of Malian djembe music. For example, in suku, a repeating twelve-pulse cycle with multiple levels of accent is set into groups of three, each of which has a non-isochronous “short-medium-long” pulse pattern [103]. In sum, despite impressive diversity, rhythms from around the world are characterized by a restricted tempo range, multi-layered patterning, accenting, and predictability.

Further evidence that rhythm relies on conserved biology comes from the fact that the acoustic stimulus, taken alone, is often an insufficient basis for direct derivations of pulse and meter. Instead, these core aspects of rhythm depend on the interaction of sonic events and the brain [100, 101]. Multiple lines of evidence indicate that humans possess specialized neural mechanisms that reflexively identify and reinforce temporal regularity in sequential auditory stimuli. These mechanisms (described in greater detail below) are specialized in that they are common to most humans but apparently rare among other animals. Individuals from many species can be trained to move in reaction to a pulse, but human movements are shifted forwards in time to anticipate, rather than lag behind, upcoming events [108]. We also synchronize flexibly, easily adjusting to tempo changes that disrupt or defeat synchrony in experiments with other species (parrots represent an interesting exception) [40].

More evidence of specialization comes from our curious tendency to spontaneously impose accenting on acoustic sequences that lack it. For example, we are apt to hear alternation or triplets in sequences of physically identical events, a perceptual imposition that can be differentiated electroencephalographically [109]. A final piece of evidence for specialized neural mechanisms in human rhythm perception is the global popularity of *syncopation*, especially in dance music [110–112]. Syncopation balances anticipation, built from sounds occurring on-the-pulse, against its systematic violation by sounds occurring off-the-pulse [113]. Perceiving syncopation thus depends on a conserved ability to form an internal model of regular temporal structure that is strong enough to withstand substantial ill-fitting sonic data [111]. Together, these and other broadly conserved aspects of rhythm indicate a strong foundation in our shared biology.

### Biological foundations of rhythm

To model the biology underlying rhythm, music scientists have developed Neural Resonance Theory (NRT), the central tenet of which is that rhythm perception depends on endogenous oscillations in neural circuitry [97, 114–116]. NRT holds that such oscillations spontaneously entrain to stimulus-evoked neural responses to modulate receptivity, prediction, and motor reactivity, thus providing a mechanistic basis for pulse and meter. While this “resonant” capacity is maximally engaged by music, its primary utility appears to be in processing spoken language, which, despite being less temporally regular than music, is still sufficiently regular (between 2–8 Hz [120–480 bpm] [102]) for entrained oscillations to aid in parsing phonemes, syllables, and phrases [117, 118]. This implies that rhythm perception is intimately linked to vocal communication, just like tone perception.

A related aspect of NRT is that neural activity in auditory cortices readily couples with neural activity in parts of the brain that regulate movement, especially cortical areas and subcortical structures involved in motor planning, such as the supplementary motor and premotor cortices, the dorsal striatum, and the cerebellum [119–123] (Fig. 2C). Activity in these parts of the brain increases in response to rhythm, even in the absence of movement [122], suggesting that auditory-motor interaction may be essential to rhythm perception. The link between rhythm and movement has also been explored in studies of *groove*, a psychological concept defined by variation in the degree to which a musical stimulus inspires movement. People generally agree about degrees of groove in music [124, 125], with research suggesting a basis in common acoustical and structural features of rhythm, such as emphasized low-frequency energy (“bass”) [126, 127] and moderate levels of syncopation [111, 112, 127, 128]. Notably, groove is broadly associated with positive affect [111, 125, 129, 130], making it directly relevant to mental health.

### Applications of rhythm in mental health

So far, the clinical value of NRT has mainly been studied in the context of music therapies aimed at improving sensory and motor functions [131] (including speech [132]). However, even in these contexts, mental health benefits are often apparent. For example, in a 2021 meta-analysis of 17 randomized controlled studies testing musical interventions in Parkinson’s disease, a sub-analysis of 8 studies with mental health measures found significant benefits for mood, motivation, and emotional well-being in music conditions compared to standard care (SMD = 0.38, *N* = 273 patients) [133]. Positive mental health outcomes have also been observed in response to receptive music therapy after stroke [134, 135]. For example, one widely-cited study found that listening to music for at least one hour per day over a two-month period significantly lowered self-reported depression at 3 months post-stroke, as compared to standard medical care and rehabilitation [136]. Intriguingly, this study also reported benefits of music listening for cognitive function (memory and attention) in a well-controlled comparison to audio-book listening [136].

The capacity of rhythm to entrain activity in broad auditory-motor networks and simultaneously increase positive affect can also be hypothesized to account for a significant proportion of the benefits of musical treatments for anxiety and depression (see Section “Applications of tonality in mental health”). Specifically, engaging these networks with high-groove rhythms may provide an efficient way to disrupt maladaptive patterns of brain activity associated with negative affect and self-focused negative rumination [137–139]. Related to this hypothesis, there is growing evidence that groove is important for understanding the effects of music on cognition, particularly in the context of repetitive effortful work, which can often generate negative affect [135, 140–145]. For example, in one recent study, listening to a high-groove drum loop for just 3 min was found to be more effective than noise at improving performance on a subsequent repetitive behavioral task measuring context-dependent response inhibition (a “Stroop” test). This effect of rhythm was specific to participants who reported enjoying the drum loop and its groove. These participants also exhibited significantly greater (dorsolateral) prefrontal cortical activity during the Stroop test in the drum-loop condition, as measured using functional Near Infra-Red Spectroscopy [141].

Experimental evidence for positive effects of rhythm on certain types of cognition accords with longstanding evidence from ethnographic literature. Specifically, rhythmic music has often been used to positively transform the experience of work otherwise experienced as negative and draining (e.g., harvesting food, military drills, and moving cargo) [145, 146]. Similarly, musicians commonly experience “being in the groove” as a pleasant state of focus that offsets burdens associated with extended periods of high level performance (e.g., on tour) [125, 129, 147]. Such effects can be understood as rhythmically-driven increases in motivation and effort [143], potentially reflecting increased engagement of key cortico-basal ganglia-thalamo-cortical loop circuitry (see Fig. 2B). They are particularly well-characterized in the context of physical exercise, where music can increase enjoyment and reduce perceived exertion [148], but such benefits may also extend to less muscular tasks (see discussion of the Mozart effect in Section “Another crest in the music and health hype cycle?”). In sum, the biological foundations of rhythm provide insight into how music can be applied to address challenges in mental health associated with mood, cognition, and motivation.

## Reward

### Music and brain reward circuitry

While the framework described so far is based on an analytic separation of tonality and rhythm, the health applications of several other core elements of musicality are better considered in terms of music as a whole. Perhaps the best example is our fundamental attraction to music, as reflected in its marked capacities to stimulate wanting, liking, and learning. Over the past several decades, neuroimaging studies have demonstrated that taking pleasure in music is closely associated with activity in classical brain reward circuitry [26, 149], including the mesolimbic dopamine pathway between the ventral tegmental area (VTA) and the nucleus accumbens (NAc) [4]. Early studies used positron emission tomography with the radiolabeled dopamine D_2_ receptor ligand, [^11^C]raclopride, to show that musical *frisson* [150]*—*moments of peak neural excitement, piloerection, and “chills” that occur during music listening—are associated with surges in dopamine binding within the NAc [151, 152]. Additional evidence that music stimulates mesolimbic reward comes from functional magnetic resonance imaging studies showing, for example, that the magnitude of an individual’s NAc response to music correlates with their subjective liking for it [153].

**Fig. 3:**
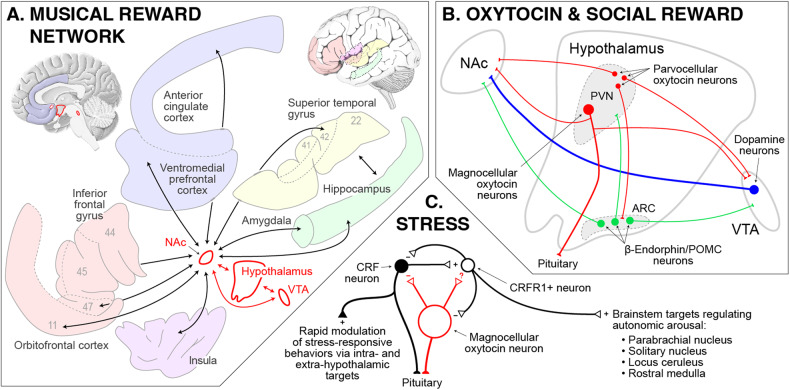
Musical reward and hypothesized relation to social connection. **A** A model of the extended musical reward network including the tripartite core (red outline) and associated cortical areas and subcortical structures (gray outline). Arrows indicate significant positive temporal correlation in blood-oxygenation-level-dependent activity between the indicated areas during pleasurable music listening. Numbers refer to Brodmann areas (**B**) A close-up of the tripartite core showing dopaminergic (blue), opioideric (green), and oxytocinergic (red) circuitry hypothesized to underpin music’s capacity to stimulate social connection. In rodent models (on which this panel is based) the derivation of reward from positive social interaction requires the oxytocinergic projections from the PVN to the NAc and VTA. **C** Interactions within the PVN between oxytocin and CRF. Oxytocin decreases the excitability of CRF neurons in mouse hypothalamic slices, and may further inhibit CRF release by modulating CRFR1-positive neurons. Note that music may also have effects on CRF that are independent of oxytocin. ARC arcuate nucleus, CRFR1 CRF receptor type 1, NAc nucleus accumbens, POMC proopiomelanocortin, PVN paraventricular nucleus, VTA ventral tegmental area.

At the level of brain networks, functional neuroimaging studies have also found that the time-course of musically-stimulated NAc activity is tightly coupled with that of activity in the VTA and hypothalamus [154]. This has led to the proposal of a “tripartite network” at the core of musical reward, with the hypothalamic node linking desire and pleasure to autonomic and neuroendocrine effects (Fig. 3A) [128, 154, 155]. Beyond this core, musical reward also engages an extended network of brain areas including the auditory, frontal, and insular cortices, as well as the amygdala and hippocampus, all of which also exhibit temporal coupling with the NAc during music listening [149, 153, 154]. These extended connections are presumed to situate musical reward with respect to sensory, integrative, somatic, affective, and memory-based aspects of musical responding, respectively.

Lastly, as in the processing of other rewarding stimuli like food, sex, and drugs, the hedonic aspects of musical reward are partially dependent on opioidergic mechanisms. This has been shown pharmacologically, as treatment with the (predominantly μ-) opioid receptor antagonists naloxone and naltrexone significantly reduces pleasure in response to musical stimuli [156, 157]. Thus, although the work described in this section has been carried out almost entirely with “Western” listeners, the results, taken together with the widespread enjoyment of music around the world, strongly support the sensitivity of brain reward circuitry to musical stimulation as a third core element of musicality.

### Applications of musical reward in mental health

In keeping with the central importance of reward in our everyday lives, this element of musicality has extremely broad implications for mental health. Dysfunction in brain reward circuitry contributes to a wide range of psychopathology, including mood disorders, anxiety disorders, substance use disorders, eating disorders, obsessive-compulsive disorders, attention-deficit/hyperactivity disorder, autism spectrum disorders, conduct disorder, Tourette’s syndrome [158], and schizophrenia. This suggests that the benefits of many current musical treatments may be attributable to normalizing effects of tonality and rhythm on otherwise aberrant activity in brain reward circuitry. Thus, in addition to effects on core dimensions of mental health (e.g., anxiety, mood, cognition, and motivation), musical treatments have also been found efficacious in more specific cases of psychopathology that specifically feature reward dysfunction. Some examples include: substance-use disorder, where adding music therapy to standard treatment can improve motivation to rehabilitate and abstinence [159]; anorexia nervosa, where interactive music therapy can stimulate reductions in post-meal anxiety that exceed those of other treatments [160]; and Tourette’s syndrome, where music listening, performance, and even imagined performance, can be an effective tic suppressant [161].

Further evidence of music’s efficacy against reward-related dysfunction comes from treatments applied to prominent transdiagnostic symptoms, like fatigue [162], apathy [163, 164], and anhedonia [165]. For example, in a study of nursing home residents age 60+ with mild-to-moderate dementia, a twelve-week interactive music therapy intervention significantly reduced apathy and improved communication, in comparison with a treatment-as-usual control [163]. The effect sizes were relatively small (SMD = 0.32 and 0.15 respectively), but given the central importance of apathy in dementia and other psychopathology [166–168], they represent an important starting point for further investigation. In sum, the capacity of music to modulate brain reward circuitry provides a strong mechanistic basis for its benefits across a wide variety of functional disorders in mental health. A better understanding of how and when music stimulates reward is thus critical to advancing music’s therapeutic benefits for mental health.

## Sociality

### Synchrony

Converging evidence indicates that engaging in music with other people is an effective way to stimulate interpersonal affiliation and social connection [44]. Psychological experiments, for example, have repeatedly shown that interpersonal temporal coordination (or “synchrony”) in behavior—a defining feature of musical interaction—strengthens social bonds between participants. This has been measured in terms of increased feelings of affiliation and self-other similarity [169, 170], trust behaviors in economic games [171, 172], and real-world cooperation [173–177] (reviewed in [178]). Another line of evidence comes from physiological experiments showing that recreational forms of behavioral synchrony—e.g., in group singing, drumming, or exercise—can upregulate oxytocin secretion [155, 179–182], downregulate cortisol secretion [155, 181, 183–185], modulate immune reactivity [182, 184, 185], and decrease pain [186, 187].

In addition to behavioral synchrony, music almost certainly facilitates affiliation and social connection through inducing synchrony in affect. This is perhaps best illustrated by the *Iso Principle* for mood management in music therapy, one of a few core methods that remains consistent across diverse approaches and therapeutic goals [188]. Iso Principle is the practice of initiating treatment sessions with music that is parameterized to match the patient’s current mood, creating a basis of shared affect that can then be leveraged to shift mood through musical changes. While the neural basis of synchrony’s effects on social neurobiology has yet to be studied in detail (see [189] for leading hypotheses), at a psychological level it appears to work through empathetic processes that increase trust and promote openness to further interaction and direction [190].

A final line of evidence comes from ethnographic and historical observations indicating that music (and dance) are commonly associated with contexts involving high levels of social cohesion. Major examples include religious rituals, cooperative labor, and military drill, as well as overt expressions of group solidarity like political chants, football songs, and national anthems [145, 146]. Taken together, these findings strongly support the sensitivity of neural mechanisms supporting affiliation and social connection to musical stimulation as a fourth core element of musicality.

### Oxytocin and social reward

Although many artistic and aesthetic experiences are capable of eliciting intense pleasure, music stands out for the regularity with which it does so [157]. Research suggests that frisson, for example, are induced by music at about four times the rate that they are induced by other stimuli, including the visual arts and literature combined [191]. This begs the question of why music is so rewarding.

A potential hint comes from the fact that frisson are also induced at high rates by inspirational speech [191, 192]. From a mechanistic perspective, this can be taken as support for the hypothesis that the reward potency of music (and speech) reflects high temporal predictability relative to other artistic stimuli [150, 153], which is particularly well-suited to anticipatory aspects of reward processing [193]. At the same time, phylogenetic and developmental perspectives have given rise to the hypothesis that the reward potency of music reflects its basis in social communication [149]. In this non-mutually exclusive view, music’s capacity to stimulate reward processing also reflects the activity of evolved neural mechanisms that develop to afford the voice with major modulatory control over the rewards of social interaction.

Interest in the link between music and social reward has led many researchers to posit a role for the hypothalamic neuropeptide oxytocin in musicality [5, 44, 149, 194, 195], following on its essential functions in affiliative behavior and social bonding (Fig. 3B) [196–200]. More specifically, music can be hypothesized to stimulate endogenous oxytocin mechanisms that upregulate dopaminergic (and related opioidergic) aspects of reward processing [198], thereby increasing sensitivity to musical rewards in social context. An important corollary of this hypothesis also addresses the anti-stress effects of music [201], as music-induced oxytocin release in the hypothalamus may also modulate local corticotropin releasing factor (CRF) circuitry to downregulate activity in the hypothalamic-pituitary-adrenal axis and the sympathetic division of the autonomic nervous system (Fig. 3C) [202–206].

### Applications of sociality in mental health

Social functioning—as reflected in the structure, function, and quality of an individual’s social connections—is a critical determinant of mental health in patients across prominent psychiatric disorders [207, 208] as well as the general public [209, 210]. This implies that effects of musical treatment of the neurobiology of social functioning may be of even broader significance than closely related effects on brain reward circuitry. However, before describing the clinical evidence supporting such effects, it should be noted that the extent to which musical treatment must involve live interaction to impact social neurobiology is presently unclear. Sound recording is only 160 years old, which implies that the vast majority of our collective experience with music has occurred in social contexts. Accordingly, there is an important sense in which listening to recorded music, even alone, may remain inherently social in neurobiological terms. Our attribution of recorded music to a person (or people) with communicative intent is essentially reflexive [211], particularly when it comprises vocals. It is also clear that recorded music is often a potent stimulus for behavioral and affective synchrony. Thus, listening to music alone may stimulate social neurobiology in many of the same ways as live musical interaction. Nevertheless, until shown otherwise, it seems reasonable to assume that live interaction is the more potent stimulus for leveraging music’s effect on sociality (e.g., see [212–214]).

Operationally, social functioning is targeted by interactive approaches to music therapy designed to support interpersonal responding, coordination, and synchrony [11, 215]. A large body of evidence supports the benefits of such approaches in autism spectrum disorders [216–221]. Some of this evidence is summarized in a 2022 meta-analysis of 26 controlled studies including 1,165 children with diagnoses of an autism spectrum disorder (ranging from mild to severe). This analysis compared music therapy to non-musical standard care or a “placebo” therapy over an average duration of 2.5 months (SD = 2.0), with session frequency varying from daily to weekly in shorter and longer studies respectively [216]. Directly after the intervention, significant benefits associated with music therapy included improvement in clinical global impression (risk ratio=1.22, 8 studies, 583 patients), reduced total autism symptom severity (SMD = 0.83, 9 studies, 575 patients), and better quality of life for clients and/or their families (SMD = 0.28, 3 studies, 340 patients). During the intervention, music therapy was also associated with significant improvements in non-verbal communication (SMD = 1.06, 3 studies, 50 patients) and behavioral adaptation (SMD = 1.19, 4 studies, 52 patients); in the 1–5 months following the intervention, music therapy was associated with reduced total autism symptom severity (SMD = 0.93, 2 studies, 69 patients) and improved self-esteem (SMD = 0.86, 1 study, 35 patients) [216]. For context, the overall SMD for autism interventions based on Applied Behavior Analysis (a common non-musical behavioral therapy) has been estimated at 0.36 for treating general autism symptoms (based on 14 studies with 555 patients) [222].

Further evidence supporting the benefits of music therapy for social functioning comes from studies on schizophrenia [223]. A 2020 meta-analysis of 15 controlled studies involving 964 adults diagnosed with schizophrenia or a schizophrenia-like disorder highlighted significant improvements in negative symptoms (such as flat affect, poor social interactions, and apathy) when adjunct interactive and/or receptive music therapy was compared to standard care (SMD = 0.56) [164]. This aligns with an earlier 2017 meta-analysis that more specifically investigated social functioning, reporting benefits from two controlled studies involving adults with schizophrenia in which music therapy was compared to antipsychotic medication (SMD = 0.72, *N* = 160 patients) [224]. For context, the SMD of antipsychotic medications for treating negative symptoms in schizophrenia has been estimated at 0.35, based on 167 studies with 28,102 patients [225].

There is also some evidence that musical interventions can impact social functioning in Alzheimer’s disease and related dementias. For example, individual studies have reported significant benefits of interactive music therapy on language functioning [226] and receptive music therapy on social engagement [227]. However, reviews and meta-analyses suggest that such social effects are mainly derivative from primary benefits that reduce agitation, anxiety, and depression [228, 229].

Finally, outside of the clinic, musical therapy has long been valued as a non-verbal path to social connection in children with special needs [215, 221], as well as a way to combat social isolation and loneliness, particularly in older adults living alone and/or with serious disease [184, 230]. In sum, music’s capacity to stimulate the neurobiology of affiliation and social connection is associated with benefits in multiple major mental health disorders and across the lifespan.

## Individual differences in musicality

Despite strong foundations in our shared biology, there is also substantial individual variation in neural sensitivity to the core elements of musicality. At the low end of the spectrum are individuals who cannot carry a tune or dance in time, some of whom find music irritating and actively avoid it [231]. Conversely, at the high end are individuals who find it difficult to live without music, some of whom create works of art that transcend their geographic and temporal horizons [232]. This high degree of individual variation in musical appreciation and engagement implies that there may also be substantial variation in individual capacity to benefit from musical treatment. In this section and the next I review research on understanding individual variation in musicality, outlining how its measurement may be used to increase the precision with which musical treatments are applied. Accordingly, I argue that better applications of music in mental health depend not only on aligning the neurophysiological effects of music’s core elements with specific clinical targets, but also on matching treatment content to individual differences in musicality.

### Psychoacoustic testing

Tests of tone and rhythm perception have long served as the primary way to measure individual differences in musicality. Performance on the most basic of these tests—e.g., measuring sensitivity to harmony and pulse—tends to be positively skewed [233], reflecting a commonplace competency for music similar to that which we possess for language [41]. Nevertheless, there is still considerable variation in basic test scores, and this is increased for tests that probe more sophisticated musical abilities [234].

### Environmental factors

Researchers have traditionally sought explanations for individual differences in musicality based on environmental factors. One of the most important environmental factors is *formal training*, a process by which individuals explicitly learn specific motor skills and rules for music performance and composition [235]. Formal training is particularly important for explaining sophisticated musical abilities, e.g., as assessed by Goldsmith’s Musical Sophistication Index (Gold-MSI) [234]. Another important environmental factor is *musical enculturation*, i.e., the process of implicitly learning the statistical properties of the music to which one is developmentally exposed. Many studies have demonstrated effects of training and enculturation on psychoacoustic tests (e.g. [236, 237]). Though sometimes framed as evidence against biological constraints, such effects may be better considered in terms of how biological constraints manifest in the face of environmental variation [56, 78].

### Biological factors

Progress is also being made towards understanding the genetic basis of musicality [27]. Early work provided evidence that genetic factors explain surprising amounts of phenotypic variability in psychoacoustic test performance (e.g., 70–80% in tone perception [238]), as well as time spent practicing music (e.g., 40–70% [239]; see also [240]). More recently, genome-wide association (GWA) techniques have been applied to musicality [241–243]. The largest of these GWA studies to date has focused on rhythm perception [243]—assessed via the question “can you clap in time with a musical beat?”—in a sample of over 606,825 individuals, accessed via an academic collaboration with 23andMe, Inc. The results indicated that beat perception and synchronization depend on many genes, with variation at 69 loci spread across 20 chromosomes being significantly associated with survey responses after linkage disequilibrium pruning. Additional analyses found enriched expression of genes implicated by these loci in brain-specific regulatory elements as well as fetal brain tissue, indicating potential roles in regulating neurodevelopment. Similar analyses focused on the adult brain found enriched expression in structures implicated in rhythm and reward, including the frontal and temporal cortices, cerebellum, basal ganglia, nucleus accumbens, and hypothalamus (see Figs. 2C and 3B).

Although complex traits like our sensitivity to rhythm are expected to be polygenic [243], some studies have also focused on associations between musicality and individual genes. One of the best studied genes in this context is *AVPR1A*, which encodes the vasopressin 1A receptor, a major component of the arginine vasopressin and oxytocin signaling pathways [196, 244]. Genetic variation in the promotor region of *AVPR1A* has been associated with phenotypic variation in psychoacoustic test scores [245, 246], time spent attentively listening to music [247], and being a dancer as opposed to another type of athlete [248]. Variation in *AVPRA1* has also been associated with verbal memory [249], acoustic startle [250], amygdala activity [251], prosocial behavior [252], pair-bonding [253], and autism [254]. As intriguing as these associations are, however, it should also be noted that several studies have looked and failed to find associations between musical ability/behavior and *AVPR1A* polymorphism [242, 255]. Other genes of particular interest include *VRK2*, *FANCL*, *MAPT*, *MAPK3*, *GATA2*, *GBE1*, *GPM6A*, *PCDH7, SCL64A*, and *UGT8* among others (see [27] and [243]).

Lastly, progress in understanding the biology underlying individual differences in musicality has also come from studies of disordered music perception. *Congenital amusia* [256] is an umbrella term for lifelong deficits in music perception that prevent people from singing in tune [257], dancing in time [258], or deriving pleasure from music [259]. Deficits in tone perception (or *tone deafness*) is the best studied form of congenital amusia: it runs in families [238, 260] and is associated with decreased connectivity between the auditory cortices and the inferior frontal gyrus [261, 262], potentially reflecting abnormal frontotemporal cortical development [263]. The prevalence of tone deafness is approximately 1.5%, with as many as 4.2% of people exhibiting a lesser form of impairment [264]. Deficits in rhythms perception (or *beat deafness*) appears to be at least as common [264]. Finally the prevalence of *music-specific anhedonia*, which, as the name implies, occurs despite otherwise normal hedonic functioning, is estimated at about 5% [265].

## Hypotheses for precision medicine

Faced with questions about whether a patient is sufficiently musical to engage in treatment, many music therapists provide reassurance, as a significant part of their practice is dedicated to finding adaptive ways to leverage music’s capacities to align with individual strengths [266, 267]. While this *resource-oriented* approach has the benefit of allowing music therapists to work with almost anyone, the framework proposed here can potentially offer more systematic guidelines for determining whether a patient is likely to benefit from musical treatment. Fundamentally, patients with a history of strong engagement with music and keen sensitivity to its tonal, rhythmic, rewarding, and social elements would appear to be good candidates for musical treatment, especially if neurophysiological systems influenced by one or more core elements of musicality are implicated by their symptoms. Conversely, those patients who report disliking music, find it unrewarding, or otherwise qualify for congenital amusia, would seem to have a lower likelihood of benefiting.

In between these extremes are individuals whose specific *musicality profiles*—conceived as a series of measurements describing sensitivity to each core element of musicality—have important potential to inform decisions about treatment content. As an example, treatment for a patient with below-average tone perception, but normal sensitivity to musical reward, rhythm, and sociality could be personalized to align with their musicality profile by focusing on the neurophysiological effects of rhythm in an affiliative interactive context in which tonal elements are minimized or omitted.

### Defining musicality profiles

While measurements of underlying biology may improve assessments of individual differences in musicality in the future, current efforts must rely on psychoacoustic tests and surveys. Among the most promising for determining suitability for musical treatment is the Barcelona Music Reward Questionnaire (BMRQ) [265], a survey of 20 self-reported items that assess the degree to which an individual takes pleasure in different aspects of music. For individuals with normal scores on the BMRQ, further insight may be gained through a series of basic psychoacoustic tests, like the *scale test* and *out-of-key test* (for evaluating tone perception) and the off-beat test (for evaluating rhythm perception) from the Montreal Battery of Evaluation of Amusia (MBEA [233, 268]; see MBEMA for testing children aged 6 to 10 [269]). If a more comprehensive assessment is desired, clinicians can deploy the Gold-MSI (for musical sophistication) [234] or the computerized beat alignment test (for rhythm) [270].

Although not explicitly focused on music, it may also be useful to assess a patient’s level of social functioning and anxiety (e.g., with the Social Responsivity Scale [SRS] [271] and Liebowitz Social Anxiety Scale [LSAS] [272] respectively), as the results could inform decisions about the extent to which a musical intervention should target social functioning. Interactive music therapy can be hypothesized to be most effective in cases where social functioning and social anxiety are both low. By contrast, in cases where social anxiety (or anxiety more generally) is high, the most effective approach may instead require limiting social interaction, at least at first. In keeping with this hypothesis, interactive approaches to music therapy in dementia (where anxiety is often high) are significantly less effective than receptive approaches at reducing agitation and behavioral problems [229]. Similarly, in music therapy for autism—which is predominantly interactive—high comorbidity with anxiety disorders may help explain some of the heterogeneity in trial results (cf [273, 274].). Lastly, in cases where a patient is unable to complete surveys or perform perceptual tests due to developmental delay or cognitive impairment, interviewing caregivers about the patient’s history of music engagement and social functioning can offer valuable insights into their potential sensitivity to musical treatment.

### Idiosyncratic preferences

Beyond tailoring musical treatments to align neurophysiological effects with clinical targets and individual musicality profiles, treatments may also be customized based on individual music preferences or “taste” [275, 276]. In receptive music therapy, for example, it’s common for patients to nominate songs they like, with therapists providing oversight for alignment with therapeutic goals [89]. One major advantage of this approach is that listening to preferred music can be especially rewarding [151, 277]. This is often attributed to the familiarity of preferred music, which facilitates expectations, their fulfillment, and associated memories and emotions [150, 278, 279]. Other potential benefits of preferred music include fostering a sense of safety, enhancing engagement, and reducing stress [280–282]. However, personal memories and associations can also make the therapeutic value of preferred music difficult to control, especially if not carefully reviewed [283]. This is because what a person likes is not necessarily aligned with their therapeutic goals. A prime example is that people with depression often prefer music that maintains or exacerbates their sadness [284–286] (but see [285, 287, 288]). Accordingly, despite the benefits of preferred music, using novel or unknown music is advisable in some contexts.

Having already changed how people discover new music, algorithmic music recommendation systems may also find applications in mental health. However, the issue of mismatch between what a person likes and their treatment goals remains significant here as well. For example, listening to strongly preferred or popular music while attempting to focus tends to decrease task performance [140, 142]. In the extreme, the lifestyle associated with many forms of popular music is linked to substance abuse, risk-taking, suicide, homicide, and accidental death among practitioners [289]. This highlights the fact that engagement with music is not necessarily health-positive (cf [290–292].). In therapeutic contexts, though, there are still many cases in which tailoring musical interventions to idiosyncratic preferences can be beneficial. For example, in receptive music therapy for Alzheimer’s disease, listening to familiar, preferred music appears to carry benefits for self-awareness [293]. Similarly, in depression, preferred music is likely to be the most effective stimulus for normalizing brain affect and reward functions, provided that it has been properly vetted to avoid stimulating negative affect. Finally, when a patient has normal sensitivity to musical reward but only within a very restricted genre (e.g., from their youth [294]), or, reports enjoying music despite poor tone and rhythm perception [295], understanding their idiosyncratic preferences may be necessary to design effective treatment.

In sum, determining the therapeutic value of aligning musical treatment with idiosyncratic preferences is of central importance for musical applications in mental health. That said, progress in this kind of preference matching should be incorporated within a broader precision paradigm as advocated here, which aims to align the specific neurophysiological effects of musicality’s core elements with specific clinical targets and individual differences in associated responsivity.

## Skepticism and need

In this final section, I address several important points of skepticism regarding the premise of the biological framework presented here, i.e., the hypothesis that music can do more for mental health.

### Benefits from music to mental health are already at saturation

In addition to the effects of musical treatment described above (see Sections “Applications of tonality in mental health.”, “Applications of rhythm in mental health”, “Applications of musical reward in mental health”, & “Applications of sociality in mental health”.), there is strong evidence that people derive mental health benefits from more casual engagement with music. During the height of the COVID-19 pandemic, for example, more than half of 4,206 survey respondents reported engaging with music as a coping strategy, using it to derive reward, modulate mood, and/or reduce stress and anxiety [296]. Similar positive functions are apparent in pre-pandemic research as well (alongside more social functions) [2, 297–299]. Associations between music and healing have also been found in many cultures throughout human history, suggesting a potentially ancient relationship [300, 301]. Thus, even though music lies outside the mainstream of mental health care, many people are already using music to improve their condition.

Nonetheless, there are multiple ways in which music’s mental health benefits may be increased. First, expanding access to musical treatment is essential [302]; as stated in the introduction, music therapists in the US only have the capacity to treat 0.5% of adults with mental illness. I have argued that this necessitates standardizing and applying treatments within a biological framework. Second, the popular perception of music as entertainment needs to evolve to encompass its therapeutic benefits. Explaining musical treatments in biomedical terms and normalizing therapeutic modes of listening can facilitate this shift. Third, the balance in music education needs to pivot away from individual performance and back towards widespread attainment of basic skills (e.g., social singing and dancing, listening, reflecting, curating, etc.), with an explicit focus on developing lifelong tools for mental health and wellness [303].

### Another crest in the music and health hype cycle?

Even if one accepts that music has expandable mental health benefits, the importance of music’s potential might still seem overblown, here and elsewhere. It is worth revisiting the *Mozart effect* in this context, as an example of music’s real effects and associated hyperbolic overinterpretation. In 1993, a study published in the journal Nature reported that 10 min of listening to a spirited Mozart sonata, versus speech-based relaxation, or silence, improved performance on a subsequent spatial reasoning task [144]. After being picked up by popular press, this finding was transformed into the notion that “listening to Mozart actually makes you smarter” [304], which was subsequently used to market books and other media for benefits purportedly backed by science [305]. Backlash from the scientific community in the form of criticism and further investigation eventually came to show that the Mozart effect amounts to a relatively small but replicable performance boost that generalizes to other types of music (and speech) which stimulate enjoyment and arousal (SMD = 0.37 in meta-analyses) [143, 305, 306]. Thus, while we should remain guarded against hype surrounding claims about music’s potential benefits, the example of the Mozart effect should also remind us not to counter hype with dismissal.

### Low quality studies undermine claims of clinical value

The randomized double-blind placebo-controlled trial remains the gold standard for evidence in clinical medicine. However, this approach was primarily designed to test the efficacy of drug therapies, a history that creates problems for using it to test behavioral interventions, such as music therapy or psychotherapy [307, 308]. Central problems include: difficultly blinding patients and therapists to their assigned condition (treatment or control), designing appropriate “placebo” treatments, and perceived difficulty in standardizing treatment without jeopardizing therapeutic integrity [308, 309]. These problems are compounded in trials that rely on self- and/or clinician-reported outcomes (which is standard in much mental health research [309]). Consequently, concerns over study quality have often been cited in expressions of doubt over music’s clinical value (e.g. [302, 308]).

A quick survey of modern clinical research in music therapy shows that such criticism has been well-received. Improvements in control conditions and blinded outcome assessments have been gradually implemented and evidence from more carefully conducted trials has begun to accumulate. Over the last decade, there has also been a surge in meta-analytic syntheses of this work, most of which explicitly assess risk-of-bias alongside their conclusions, although they do not typically take the next step of adjusting effect size estimates accordingly (cf [96, 310].). Overall, bias assessments suggest that the certainty of evidence supporting benefits from musical treatment in mental health is moderate to low. Nonetheless, this level of certainty is consistent with many treatments in psychiatry [94]. The assertion that studies of musical treatment are especially suspect is thus poorly substantiated. Interested readers should consult bias assessments in these meta-analyses [93, 95, 96, 133, 164, 216, 224, 229], and review individual studies that exemplify high-quality research on musical treatments for conditions such as anxiety [311, 312], depression [313, 314], autism [274, 315], psychosis [316, 317], and dementia [318, 319].

### Mental health needs

In concluding this section, it is useful to briefly consider musical treatment in the context of current mental health needs. In 2007, mental health disorders were estimated to account for 14% of global disease burden [320]. In 2021, an estimated 22.8% of adults in the United States had a diagnosable mental illness, with 12.7% of adolescents having serious thoughts of suicide [17]. In opposition to this growing psychopathology, first-line treatments in psychiatry are often criticized for their limited effectiveness [94, 320, 321]. Quantifying this point, a 2022 meta-analytic evaluation of 3,782 clinical trials examining the most common adult mental health disorders across a total sample size of 650,514 patients estimated summary effect sizes of just 0.34 SMD for psychotherapy and 0.36 SMD for pharmacotherapy [94]. In depression, SMDs <0.88 represent changes in a patient’s presentation that are typically too small to be detected by a clinician, suggesting that the effects of standard treatments for depression commonly lack clinical significance [94, 322, 323]. A similar SMD threshold in schizophrenia is 0.73 [94, 324]. It is crucial to note that small summary effect sizes in meta-analyses are averages, and thus obscure the reality that a minority of patients have experienced clinically significant benefits under current treatments (due to poorly understood individual differences in treatment response). Nevertheless, the data at hand clearly indicate that new treatments are urgently needed [94].

It is in this context that advancing new standardized music-based interventions is important, not only because music affects core dimensions of mental health through the biology of tonality, rhythm, reward, and sociality, but because these avenues present an accessible, easy-entry, and low-risk approach to addressing problems for which we need solutions. Music is poorly conceived as a panacea. Instead, it has real effects on human neurobiological functions that feature prominently in mental illness, and thus has important potential in treating their disorder.

## Conclusion

The effects of music on mental health and wellness are drawing more attention now than ever before. Efforts to better understand music’s benefits and increase their integration into medicine are complicated by their impressive diversity and a lack of clarity regarding underlying biology. This review has addressed these challenges by synthesizing progress in music research from psychology, neuroscience, and psychiatry to create a framework for defining music’s neurophysiological effects and their clinical scope in biological terms. This framework includes four core elements of human musicality: *tonality*, based on tone perception and the bioacoustics of vocal emotional expression, with applications targeting mood and anxiety; *rhythm*, based on neural resonance, anticipation, and auditory-motor entrainment, with applications targeting mood, cognition, and motivation; *reward*, based on engagement of classic brain reward circuitry and the reinforcement of successful communication, with broad applications in stimulating positive affect and normalizing reward function; and *sociality*, based on synchrony and the neurobiology of affiliation, with broad applications in treating social dysfunction and increasing social connectedness. This framework rationalizes many observed benefits of musical treatment and provides a path towards a precision approach to increasing their impact. As the world continues to change and we face new challenges to mental health and wellness, music will continue to provide real biologically mediated relief. Understanding and leveraging this fact towards better treatments and interventions in psychiatry presents an important opportunity to diversify and improve care during times of pressing need.

## References

[1] Koelsch S (2015). Music-evoked emotions: principles, brain correlates, and implications for therapy. Ann N. Y Acad Sci.

[2] Lonsdale AJ, North AC (2011). Why do we listen to music? A uses and gratifications analysis. Br J Psychol.

[3] Kemper KJ, Danhauer SC (2005). Music as therapy. South Med J.

[4] Zatorre RJ (2015). Musical pleasure and reward: mechanisms and dysfunction. Ann N. Y Acad Sci.

[5] Greenberg DM, Decety J, Gordon I (2021). The social neuroscience of music: understanding the social brain through human song. Am Psychol.

[6] The U.S. Music Industries: Jobs & Benefits, by Robert Stoner and Jéssica Dutra of Economists Incorporated, prepared for the Recording Industry Association of America (RIAA), December 2020, available at www.riaa.com.

[7] Cheever T, Taylor A, Finkelstein R, Edwards E, Thomas L, Bradt J (2018). NIH/Kennedy Center Workshop on Music and the Brain: Finding Harmony. Neuron.

[8] Fitzpatrick F. Could music be a game-changer for the future of digital health? Forbes. August 25, 2021.

[9] Edwards E, St Hillaire-Clarke D, Frankowski DW, Finkelstein R, Cheever T, Chen WG (2023). NIH music-based intervention toolkit: music-based interventions for brain disorders of aging. Neurology.

[10] O’Kelly JW. Music therapy and neuroscience: opportunities and challenges. Voices: A World Forum Music Ther. 2016;16. https://voices.no/index.php/voices/article/view/2309.

[11] Silverman MJ (2015). Music therapy in mental health for illness management and recovery.

[12] Lin ST, Yang P, Lai CY, Su YY, Yeh YC, Huang MF (2011). Mental health implications of music: insight from neuroscientific and clinical studies. Harv Rev Psychiatry.

[13] Thaut MH, Francisco G, Hoemberg V (2021). The clinical neuroscience of music: evidence based approaches and neurologic music therapy. Front Neurosci.

[14] Altenmüller E, Schlaug G (2013). Neurobiological aspects of neurologic music therapy. Music Med.

[15] Tomaino CM (2022). Auditory cueing of pre-learned skills and role of subcortical information processing to maximize rehabilitative outcomes bridging science and music-based interventions. Healthcare.

[16] The American Music Therapy Association Workforce Analysis: A Descriptive Statistical Profile of the 2021 AMTA Membership and Music Therapy Community. Available at www.musictherapy.org.

[17] Substance Abuse and Mental Health Services Administration. Key substance use and mental health indicators in the United States: results from the 2021 national survey on drug use and health (HHS Publication No. PEP22-07-01-005, NSDUH Series H-57). Center for Behavioral Health Statistics and Quality, Substance Abuse and Mental Health Services Administration. https://www.samhsa.gov/data/report/2021-nsduh-annual-national-report. 2022.

[18] Jackson T. Caseloads of professional music therapists: a descriptive analysis [Master’s Thesis]. Saint Mary-of-the-Woods College, Saint Mary-of-the-woods, IN; 2016.

[19] National Institutes of Health. Music and health: understanding and developing music medicine. (2021). Available at: https://grants.nih.gov/grants/guide/pa-files/par-21-100.html.

[20] Conard NJ, Malina M, Münzel SC (2009). New flutes document the earliest musical tradition in southwestern Germany. Nature.

[21] Reck D (1977). Music of the whole earth.

[22] Bowling DL, Purves D (2015). A biological rationale for musical consonance. Proc Natl Acad Sci.

[23] Darwin C (1871). The descent of man and selection in relation to sex.

[24] Fitch WT, Bolhuis JJ, Everaert M (2013). Musical protolanguage: Darwin’s theory of language evolution revisited. Birdsong, speech, and language: exploring the evolution of the mind and brain.

[25] Fitch WT (2006). The biology and evolution of music: a comparative perspective. Cognition.

[26] Koelsch S (2014). Brain correlates of music-evoked emotions. Nat Rev Neurosci.

[27] Gingras B, Honing H, Peretz I, Trainor LJ, Fisher SE (2015). Defining the biological bases of individual differences in musicality. Philos Trans R Soc B Biol Sci.

[28] Albouy P, Mehr SA, Hoyer RS, Ginzburg J, Zatorre RJ. Spectro-temporal acoustical markers differentiate speech from song across cultures (preprint). bioRxiv. 2023.01.29.526133. 10.1038/s41467-024-49040-3 PMC11156671 38844457

[29] Haiduk F, Fitch WT (2022). Understanding design features of music and language: the choric/dialogic distinction. Front Psychol.

[30] Bowling DL, Purves D, Gill KZ (2018). Vocal similarity predicts the relative attraction of musical chords. Proc Natl Acad Sci.

[31] Panksepp J (2009). The emotional antecedents to the evolution of music and language. Music Sci.

[32] Wallin NL. Biomusicology: neurophysiological, neuropsychological, and evoluitonary perspectives on the orgins and purposes of music. Pendragon Press, Maestag, UK; 1991.

[33] Honing H, Honing H (2019). Musicality as an updeat to music: introduction and research agenda. Origins of Musicality.

[34] Patel A (2008). Music, language, and the brain.

[35] Dissanayake E, Wallin NL, Merker B, Brown S (2000). Antecedents of the temporal arts in early mother-infant interaction. The origins of music.

[36] Brown S, Wallin NL, Merker B, Brown S (2001). The ‘musilanguage’ model of music evolution. The origins of music.

[37] Thompson WF, Marin MM, Stewart L (2012). Reduced sensitivity to emotional prosody in congenital amusia rekindles the musical protolanguage hypothesis. Proc Natl Acad Sci USA.

[38] Mithen S (2007). The singing neaderthals: the origins of music, language, mind, and body.

[39] Hoeschele M, Merchant H, Kikuchi Y, Hattori Y, ten Cate C (2015). Searching for the origins of musicality across species. Philos Trans R Soc B Biol Sci.

[40] Patel AD (2021). Vocal learning as a preadaptation for the evolution of human beat perception and synchronization. Philos Trans R Soc B Biol Sci.

[41] Trehub S (2003). The developmental origins of musicality. Nat Neurosci.

[42] Miller G, Brown S, Merker B, Wallin C (2000). Evolution of human music through sexual selection. The origins of music.

[43] Merker B, Morley I, Zuidema W (2015). Five fundamental constraints on theories of the origins of music. Philos Trans R Soc B Biol Sci.

[44] Savage PE (2021). Music as a coevolved system for social bonding. Behav Brain Sci.

[45] Mehr SA, Krasnow MM, Bryant GA, Hagen EH (2021). Origins of music in credible signaling. Behav Brain Sci.

[46] Cross I (2001). Music, cognition, culture and evoution. Ann N. Y Acad Sci.

[47] Huron D (2001). Is music an evolutionary adaptation?. Ann N. Y Acad Sci.

[48] Trainor LJ. The origins of music: auditory scene analysis, evolution, and culture in musical creation. In: Honing H, editor. Origins of Musicality. MIT Press; 2019. p. 81–112.

[49] Rossing TD, Moore FR, Wheeler PA. The science of sound. Addison Wesley; 2002.

[50] Brown S, Jordania J (2013). Universals in the world’s musics. Psychol Music.

[51] Savage PE, Brown S, Sakai E, Currie TE (2015). Statistical universals reveal the structures and functions of human music. Proc Natl Acad Sci USA.

[52] McAdams S, Deutsch D (2013). Musical timbre perception. The Psychology of Music.

[53] Schwartz DA, Howe CQ, Purves D (2003). The statistical structure of human speech sounds predicts musical universals. J Neurosci.

[54] Negus VE. The comparative anatomy and physiology of the larynx. Hafner publishing company; 1949.

[55] Ozaki Y, et al. Globally songs are slower, higher, and use more stable pitches than speech [Stage 2 Registered Report]. Peer Community Regist. Reports 10.31234/osf.io/jr9x7. 10.1126/sciadv.adm9797 PMC11095461 38748798

[56] Bowling DL, Hoeschele M, Gill KZ, Fitch WT (2017). The nature and nurture of musical consonance. Music Percept.

[57] McBride J, Tlusty T. Cross-cultural data shows musical scales evolved to maximise imperfect fifths. 2020. 10.48550/arXiv:1906.06171v2.

[58] Gill KZ, Purves D (2009). A biological rationale for musical scales. PLoS One.

[59] Prasad VKK (2008). Ragas in Indian music.

[60] Tokita AM (1996). Mode and scale, modulation and tuning in Japanese Shamisen music: the case of Kiyomoto narrative. Ethnomusicology.

[61] Vos PG, Troost JM (1989). Ascending and descending melodic intervals: statistical findings and their perceptual relevance. Music Percept.

[62] Bowling DL, Gill K, Choi JD, Prinz J, Purves D (2010). Major and minor music compared to excited and subdued speech. J Acoust Soc Am.

[63] Bowling DL, Sundararajan J, Han S, Purves D (2012). Expression of emotion in eastern and western music mirrors vocalization. PLoS One.

[64] Von Hippel P, Huron D (2000). Why do skips precede reversals? the effect of tessitura on melodic structure. Music Percept.

[65] Bowling DL (2017). Body size and vocalization in primates and carnivores. Sci Rep.

[66] Banse R, Scherer KR (1996). Acoustic profiles in vocal emotion expression. J Pers Soc Psychol.

[67] Eerola T, Ferrer R, Alluri V (2012). Timbre and affect dimensions: evidence from affect and similarity ratings and acoustic correlates of isolated instrument sounds. Music Percept.

[68] Arnal LH, Flinker A, Kleinschmidt A, Giraud AL, Poeppel D (2015). Human screams occupy a privileged niche in the communication soundscape. Curr Biol.

[69] Tsai C (2010). Aggressiveness of the growl-like timbre: acoustic characterisctis, musical implications, and biomechanical mechanism. Music Percept.

[70] Trehub SE, Unyk AM, Trainor LJ (1993). Adults identify infant-directed music across cultures. Infant Behav Dev.

[71] Mehr SA, Singh M, York H, Glowacki L, Krasnow MM. Form and function in human song. Curr. Biol. 2018;1–13. 10.1016/j.cub.2017.12.042 PMC5805477 29395919

[72] Unyk AM, Trehub SE, Trainor LJ, Schellenberg EG (1992). Lullabies and simplicity: a cross-cultural perspective. Psychol Music.

[73] Huron D (2008). A comparison of average pitch height and interval size in major-and minor-key themes: evidence consistent with affect-related pitch prosody. Empir Musicol Rev.

[74] Bowling DL (2021). Harmonicity and roughness in the biology of tonal aesthetics. Music Percept.

[75] Terhardt E (1984). The concept of musical consonance: a link between music and psychoacoustics. Music Percept.

[76] Juslin PN, Laukka P (2003). Communication of emotions in vocal expression and music performance: different channels, same code?. Psychol Bull.

[77] Juslin PN, Juslin PN, Slobada JA (2001). Communicating emotion in music performance: a review and theoretical framework. Music and emotion: theory and research.

[78] Bowling DL (2023). Vocal similarity theory and the biology of musical tonality. Phys Life Rev.

[79] Filippi P, Congdon J, Hoang J, Bowling DL, Reber SA, Pašukonis A (2017). Humans recognize emotional arousal in vocalizations across all classes of terrestrial vertebrates: evidence for acoustic universals. Proc R Soc B Biol Sci.

[80] Gerhardt KJ, Abrams RM (2000). Fetal exposures to sound and vibroacoustic stimulation. J Perinatol.

[81] DeCasper J, Fifer WP (1980). Of human bonding: newborns prefer their mothers’ voices. Science.

[82] Trevarthen C (1999). Musicality and the intrinsic motive pulse: evidence from human psychobiology and infant communication. Music Sci.

[83] Langner G (1992). Periodicity coding in the auditory system. Hear Res.

[84] Bidelman GM, Krishnan A (2009). Neural correlates of consonance, dissonance, and the hierarchy of musical pitch in the human brainstem. J Neurosci.

[85] Feng L, Wang X (2017). Harmonic template neurons in primate auditory cortex underlying complex sound processing. Proc Natl Acad Sci USA.

[86] Tang C, Hamilton LS, Chang EF (2017). Intonational speech prosody encoding in the human auditory cortex. Science.

[87] Pisanski K, Bryant GA. The evolution of voice perception. In: Eidsheim NS, Meizel K, editors. The Oxford Handbook of Voice Studies. Oxford University Press; 2019. p. 268–300.

[88] Elfenbein HA, Laukka P, Althoff J, Chui W, Iraki FK, Rockstuhl T (2022). What do we hear in the voice? An open-ended judgment study of emotional speech prosody. Personal Soc Psychol Bull.

[89] Grocke D, Wigram T (2006). Receptive methods in music therapy: techniques and clinical applications for music therapy clinicians, educators, and students.

[90] Lin MF, Hsieh YJ, Hsu YY, Fetzer S, Hsu MC (2011). A randomised controlled trial of the effect of music therapy and verbal relaxation on chemotherapy-induced anxiety. J Clin Nurs.

[91] Lin HH, Chang YC, Chou HH, Chang CP, Huang MY, Liu SJ (2019). Effect of music interventions on anxiety during labor: a systematic review and meta-analysis of randomized controlled trials. PeerJ.

[92] Kühlmann AYR, de Rooij A, Kroese LF, van Dijk M, Hunink MGM, Jeekel J (2018). Meta-analysis evaluating music interventions for anxiety and pain in surgery. Br J Surg.

[93] Lu G, Jia R, Liang D, Yu J, Wu Z, Chen C (2021). Effects of music therapy on anxiety: a meta-analysis of randomized controlled trials. Psychiatry Res.

[94] Leichsenring F, Steinert C, Rabung S, Ioannidis JPA (2022). The efficacy of psychotherapies and pharmacotherapies for mental disorders in adults: an umbrella review and meta-analytic evaluation of recent meta-analyses. World Psychiatry.

[95] Aalbers S, Fusar-Poli L, Freeman RE, Spreen M, Ket JCF, Vink AC (2017). Music therapy for depression (review). Cochrane Database Syst Rev.

[96] Tang Q, Huang Z, Zhou H, Ye P (2020). Effects of music therapy on depression: a meta-analysis of randomized controlled trials. PLoS One.

[97] van Noorden L, Moelants D (1999). Resonance in the perception of musical pulse. J N. Music Res.

[98] Moelants D. Preferred tempo reconsidered. Proc. 7th Int. Conf. Music Percept. Cogn. 2002. p. 580–3.

[99] Bruno H (2005). Sensorimotor synchronization: a review of the tapping literature. Psychon Bull Rev.

[100] Fitch W (2013). Rhythmic cognition in humans and animals: distinguishing meter and pulse perception. Front Syst Neurosci.

[101] London J (2012). Hearing in Time.

[102] Ding N, Patel A, Chen L, Butler H, Luo C, Poeppel D (2017). Temporal modulations in speech and music. Neurosci Biobehav Rev.

[103] Polak R (2010). Rhythmic feel as meter: non-isochronous beat subdivision in jembe music from mali. Music Theory Online.

[104] Polak R, London J, Jacoby N (2016). Both isochronous and non-isochronous metrical subdivision afford precise and stable ensemble entrainment: a corpus study of malian jembe drumming. Front Neurosci.

[105] Polak R. Non-isochronous meter is not irregular, a review of theory and evidence. In: Gegliederte Zeit: 15. Jahreskongress def Gesellschaft für Musiktheorie 2020, GMTH, Olms, DE; p. 365–79.

[106] Roddy D (2007). The evolution of blast beats.

[107] Clayton M (2020). Theory and practice of long-form non-isochronous meters, the case of the North Indian rupak tal. Music Theory Online.

[108] Patel A (2014). The evolutionary biology of musical rhythm: was Darwin wrong?. PLoS Biol.

[109] Iversen JR, Repp BH, Patel AD (2009). Top-down control of rhythm perception modulates early auditory responses. Ann N. Y Acad Sci.

[110] Snoman R. Dance music manual: tools, toys, and techniques. Focal Press; 2019.

[111] Witek MAG, Clarke EF, Wallentin M, Kringelbach ML, Vuust P (2014). Syncopation, body-movement and pleasure in groove music. PLoS One.

[112] Witek MAG (2020). A critical cross-cultural study of sensorimotor and groove responses to syncopation among Ghanaian and American university students and staff. Music Percept.

[113] Longuet-Higgins HC, Lee CS (1984). The rhythmic interpretation of monophonic music. Music Percept Interdiscip J.

[114] Giraud AL, Poeppel D (2012). Cortical oscillations and speech processing: emerging computational principles and operations. Nat Neurosci.

[115] Doelling KB, Florencia Assaneo M, Bevilacqua D, Pesaran B, Poeppel D (2019). An oscillator model better predicts cortical entrainment to music. Proc Natl Acad Sci USA.

[116] Large EW, Snyder JS (2009). Pulse and meter as neural resonance. Ann N. Y Acad Sci.

[117] Poeppel D, Assaneo MF (2020). Speech rhythms and their neural foundations. Nat Rev Neurosci.

[118] Ding N, Melloni L, Zhang H, Tian X, Poeppel D (2015). Cortical tracking of hierarchical linguistic structures in connected speech. Nat Neurosci.

[119] Grahn JA, Brett M (2007). Rhythm and beat perception in motor areas of the brain. J Cogn Neurosci.

[120] Grahn JA, Rowe JB (2009). Feeling the beat: premotor and striatal interactions in musicians and nonmusicians during beat perception. J Neurosci.

[121] Chen JL, Zatorre RJ, Penhune VB (2006). Interactions between auditory and dorsal premotor cortex during synchronization to musical rhythms. Neuroimage.

[122] Chen JL, Penhune VB, Zatorre RJ (2008). Listening to musical rhythms recruits motor regions of the brain. Cereb Cortex.

[123] Kotz SA, Brown RM, Schwartze M (2016). Cortico-striatal circuits and the timing of action and perception. Curr Opin. Behav Sci.

[124] Madison GS (2006). Experiencing groove induced by music: consistency and phenomenology. Music Percept.

[125] Janata P, Tomic ST, Haberman JM (2012). Sensorimotor coupling in music and the psychology of the groove. J Exp Psychol Gen.

[126] Hove MJ, Marie C, Bruce IC, Trainor LJ (2014). Superior time perception for lower musical pitch explains why bass-ranged instruments lay down musical rhythms. Proc Natl Acad Sci.

[127] Stupacher J, Hove MJ, Janata P (2016). Audio features underlying perceived groove and sensorimotor synchronization in music. Music Percept Interdiscip J.

[128] Bowling DL, Graf Ancochea P, Hove MJ, Fitch WT (2019). Pupillometry of groove: evidence for noradrenergic arousal in the link between music and movement. Front Neurosci.

[129] Berliner PF (1994). Thinking in jazz: the infinite art of improvisation.

[130] Matthews TE, Witek MAG, Lund T, Vuust P, Penhune VB (2020). The sensation of groove engages motor and reward networks. Neuroimage.

[131] Dalla Bella S. The use of rhythm in rehabilitation for patients with movement disorders. Music Aging Brain 2020;383–406.

[132] Tomaino CM (2012). Effective music therapy techniques in the treatment of nonfluent aphasia. Ann N. Y Acad Sci.

[133] Zhou Z, Zhou R, Wei W, Luan R, Li K (2021). Effects of music-based movement therapy on motor function, balance, gait, mental health, and quality of life for patients with Parkinson’s disease: a systematic review and meta-analysis. Clin Rehabil.

[134] Yoo GE, Kim SJ (2016). Rhythmic auditory cueing in motor rehabilitation for stroke patients: systematic review and meta-analysis. J Music Ther.

[135] Sihvonen AJ (2017). Music-based interventions in neurological rehabilitation. Lancet Neurol.

[136] Särkämö T (2008). Music listening enhances cognitive recovery and mood after middle cerebral artery stroke. Brain.

[137] Hamilton JP, Farmer M, Fogelman P, Gotlib IH (2015). Depressive rumination, the default-mode network, and the dark matter of clinical neuroscience. Biol Psychiatry.

[138] Moberly NJ, Watkins ER (2008). Ruminative self-focus and negative affect: an experience sampling study. J Abnorm Psychol.

[139] Boehme S, Miltner WHR, Straube T (2014). Neural correlates of self-focused attention in social anxiety. Soc Cogn Affect Neurosci.

[140] Orpella J, Bowling DL, Tomaino C, Ripollés P. Affectively parameterized music improves mood and attention (preprint). PsychiArXiv. 2023. 10.31234/osf.io/yauxt

[141] Fukuie T (2022). Groove rhythm stimulates prefrontal cortex function in groove enjoyers. Sci Rep.

[142] Huang RH, Shih YN (2011). Effects of background music on concentration of workers. Work.

[143] Thompson WF, Schellenberg EG, Husain G (2001). Arousal, mood, and the Mozart effect. Psychol Sci.

[144] Rauscher FH, Shaw GL, Ky KN (1993). Music and spatial task performance. Nature.

[145] Gioia T (2006). Work songs.

[146] McNeill W (1995). Keeping together in time.

[147] Dietrich A (2004). Neurocognitive mechanisms underlying the experience of flow. Conscious Cogn.

[148] Schaffert N, Janzen TB, Mattes K, Thaut MH (2019). A review on the relationship between sound and movement in sports and rehabilitation. Front Psychol.

[149] Koelsch S (2020). A coordinate-based meta-analysis of music-evoked emotions. Neuroimage.

[150] Huron D, Margulis EH. Musical expectancy and thrills. In: Juslin PN, Sloboda JA, editors. Handbook of music and emotion: theory, research, applications. Oxford University Press; 2010. p. 575–604.

[151] Salimpoor VN, Benovoy M, Larcher K, Dagher A, Zatorre RJ (2011). Anatomically distinct dopamine release during anticipation and experience of peak emotion to music. Nat Neurosci.

[152] Blood AJ, Zatorre RJ (2001). Intensely pleasurable responses to music correlate with activity in brain regions implicated in reward and emotion. Proc Natl Acad Sci USA.

[153] Salimpoor VN (2013). Interactions between the nucleus accumbens and auditory cortices predict music reward value. Science.

[154] Menon V, Levitin DJ (2005). The rewards of music listening: response and physiological connectivity of the mesolimbic system. Neuroimage.

[155] Bowling DL (2022). Endogenous oxytocin, cortisol, and testosterone in response to group singing. Horm Behav.

[156] Mallik A, Chanda ML, Levitin DJ (2017). Anhedonia to music and mu-opioids: evidence from the administration of naltrexone. Sci Rep.

[157] Goldstein A (1980). Thrills in response to music and other stimuli. Physiol Psychol.

[158] Dichter GS, Damiano CA, Allen JA (2012). Reward circuitry dysfunction in psychiatric and neurodevelopmental disorders and genetic syndromes: animal models and clinical findings. J Neurodev Disord.

[159] Wu Q (2020). Effectiveness of music therapy on improving treatment motivation and emotion in female patients with methamphetamine use disorder: a randomized controlled trial. Subst Abus.

[160] Bibb J, Castle D, Newton R (2015). The role of music therapy in reducing post meal related anxiety for patients with anorexia nervosa. J Eat Disord.

[161] Bodeck S, Lappe C, Evers S (2015). Tic-reducing effects of music in patients with Tourette’s syndrome: self-reported and objective analysis. J Neurol Sci.

[162] Atkinson TM (2020). Association between music therapy techniques and patient-reported moderate to severe fatigue in hospitalized adults with cancer. JCO Oncol Pr.

[163] Tang Q (2018). Effect of music intervention on apathy in nursing home residents with dementia. Geriatr Nurs.

[164] Jia R (2020). The effectiveness of adjunct music therapy for patients with schizophrenia: a meta‐analysis. Psychiatry Res.

[165] Janzen TB, Al Shirawi MI, Rotzinger S, Kennedy SH, Bartel L (2019). A pilot study investigating the effect of music-based intervention on depression and anhedonia. Front Psychol.

[166] Skorvanek M (2015). The associations between fatigue, apathy, and depression in Parkinson’s disease. Acta Neurol Scand.

[167] Bortolon C, Macgregor A, Capdevielle D, Raffard S (2018). Apathy in schizophrenia: a review of neuropsychological and neuroanatomical studies. Neuropsychologia.

[168] Lechowski L (2009). Persistent apathy in Alzheimer’s disease as an independent factor of rapid functional decline: the REAL longitudinal cohort study. Int J Geriatr Psychiatry.

[169] Hove MJ, Risen JL (2009). It’s all in the timing: interpersonal synchrony increases affiliation. Soc Cogn.

[170] Reddish P, Fischer R, Bulbulia J (2013). Let’s dance together: synchrony, shared intentionality and cooperation. PLoS One.

[171] Anshel A, Kipper DA (1988). The influence of group singing on trust and cooperation. J Music Ther.

[172] Wiltermuth SS, Heath C (2009). Synchrony and cooperation. Psychol Sci.

[173] Valdesolo P, Desteno D (2011). Synchrony and the social tuning of compassion. Emotion.

[174] Kokal I, Engel A, Kirschner S, Keysers C (2011). Synchronized drumming enhances activity in the caudate and facilitates prosocial commitment-if the rhythm comes easily. PLoS One.

[175] Cirelli L, Einarson K, Trainor L (2014). Interpersonal synchrony increases prosocial behavior in infants. Dev Sci.

[176] Cirelli L, Wan S, Trainor L (2014). Fourteen-month-old infants use interpersonal synchrony as a cue to direct helpfulness. Philos Trans R Soc Lond B Biol Sci.

[177] Kirschner S, Tomasello M (2010). Joint music making promotes prosocial behavior in 4-year-old children. Evol Hum Behav.

[178] Rennung M, Göritz AS (2016). Prosocial consequences of interpersonal synchrony: a meta-analysis. Z Psychol.

[179] Keeler JR, Roth EA, Neuser BL, Spitsbergen JM, Waters DJM, Vianney J (2015). The neurochemistry and social flow of singing: bonding and oxytocin. Front Hum Neurosci.

[180] Kreutz G (2014). Does singing facilitate social bonding?. Music Med.

[181] Good A, Russo FA (2021). Changes in mood, oxytocin, and cortisol following group and individual singing: A pilot study. Psychol Music.

[182] Grape C, Sandgren M, Hansson L, Ericson M, Theorell T (2003). Does singing promote well-being?: An empirical study of professional and amateur singers during a singing lesson. Integr Physiol Behav Sci.

[183] Schladt TM, Normann GC, Emilius R, Kudielka BM, de Jong TR, Neumann ID (2017). Choir versus solo singing: effects on mood, and salivary oxytocin and cortisol concentrations. Front Hum Neurosci.

[184] Fancourt D (2016). Singing modulates mood, stress, cortisol, cytokine and neuropeptide activity in cancer patients and carers. Ecancermedicalscience.

[185] Fancourt D, Perkins R, Ascenso S, Atkins L, Kilfeather S, Carvalho L (2016). Group drumming modulates cytokine response in mental health services users: a preliminary study. Psychother Psychosom.

[186] Fritz TH, Bowling DL, Contier O, Grant J, Villringer A (2018). Musical agency during physical exercise decreases pain. Front Psychol.

[187] Cohen E, Ejsmond-Frey R, Knight N, Dunbar R (2010). Rowers’ high: behavioural synchrony is correlated with elevated pain thresholds. Biol Lett.

[188] Davis WB (2003). Ira Maximilian Altshuler: psychiatrist and pioneer music therapist. J Music Ther.

[189] Bonini L, Rotunno C, Arcuri E, Gallese V (2022). Mirror neurons 30 years later: implications and applications. Trends Cogn Sci.

[190] Watson JC, Greenberg LS, Decety J, Ickes W (2009). Empathic resonance: a neuroscience perspective. The social neuroscience of empathy.

[191] Schurtz DR, Blincoe S, Smith RH, Powell CAJ, Combs DJY, Kim SH (2012). Exploring the social aspects of goose bumps and their role in awe and envy. Motiv Emot.

[192] Benedek M, Kaernbach C (2011). Physiological correlates and emotional specificity of human piloerection. Biol Psychol.

[193] Schultz W, Tremblay L, Hollerman JR (2000). Reward processing in primate orbitofrontal cortex and basal ganglia. Cereb Cortex.

[194] Freeman W, Wallin NL, Merker B, Brown S (2000). A neurobiological role of music in social bonding. The origins of music.

[195] Harvey AR (2020). Links between the neurobiology of oxytocin and human musicality. Front Hum Neurosci.

[196] Insel TR (2010). The challenge of translation in social neuroscience: a review of oxytocin, vasopressin, and affiliative behavior. Neuron.

[197] Dölen G, Darvishzadeh A, Huang KW, Malenka RC (2013). Social reward requires coordinated activity of nucleus accumbens oxytocin and serotonin. Nature.

[198] Itskovitch E, Bowling DL, Garner JP, Parker KJ (2022). Oxytocin and the social facilitation of placebo effects. Mol Psychiatry.

[199] Ross HE, Young LJ (2009). Oxytocin and the neural mechanisms regulating social cognition and affiliative behavior. Front Neuroendocrinol.

[200] Hung LW, Neuner S, Polepalli JS, Beier KT, Wright M, Walsh JJ (2017). Gating of social reward by oxytocin in the ventral tegmental area. Science.

[201] Linnemann A, Ditzen B, Strahler J, Doerr JM, Nater UM (2015). Music listening as a means of stress reduction in daily life. Psychoneuroendocrinology.

[202] Jamieson BB, Nair BB, Iremonger KJ (2017). Regulation of hypothalamic corticotropin-releasing hormone neurone excitability by oxytocin. J Neuroendocrinol.

[203] Winter J, Jurek B (2019). The interplay between oxytocin and the CRF system: regulation of the stress response. Cell Tissue Res.

[204] Jiang Z, Rajamanickam S, Justice NJ (2018). Local corticotropin-releasing factor signaling in the hypothalamic paraventricular nucleus. J Neurosci.

[205] Rajamanickam S, Justice NJ. Hypothamalic corticotropin-releasing factor neurons modulate behavior, endocrine, and autonomic stress responses via direct synpatic projections. Curr Opin Endocr Metab Res. 2022;100400. 10.1016/j.coemr.2022.100400.

[206] Deussing JM, Chen A (2018). The corticotropin-releasing factor family: physiology of the stress response. Physiol Rev.

[207] Porcelli S, Kasper S, Zohar J, Souery D, Montgomery S, Ferentinos P (2020). Social dysfunction in mood disorders and schizophrenia: clinical modulators in four independent samples. Prog Neuro-Psychopharmacol Biol Psychiatry.

[208] Hedley D, Uljarević M, Foley KR, Richdale A, Trollor J (2018). Risk and protective factors underlying depression and suicidal ideation in Autism Spectrum Disorder. Depress Anxiety.

[209] Holt-Lunstad J, Smith TB, Layton JB (2010). Social relationships and mortality risk: A meta-analytic review. PLoS Med.

[210] Hakulinen C, Pulkki-Råback L, Virtanen M, Jokela M, Kivimäki M, Elovainio M (2018). Social isolation and loneliness as risk factors for myocardial infarction, stroke and mortality: UK Biobank cohort study of 479 054 men and women. Heart.

[211] Launay J (2015). Musical sounds, motor resonance, and detectable agency. Empir Musicol Rev.

[212] Kragness HE, Eitel MJ, Anantharajan F, Gaudette-LeBlanc A, Barazowska B, Cirelli L (2015). An itsy bitsy audience: live performance facilitates infants’ attention and heart rate synchronization. Psychol Aesthet Creat Arts.

[213] Onderdijk KE, Swarbrick D, van Kerrebroeck B, Mantei M, Vuoskoski JK, Maes PJ (2021). Livestream experiments: the role of COVID-19, agency, presence, and social context in facilitating social connectedness. Front Psychol.

[214] Swarbrick D, Seibt B, Grinspun N, Vuoskoski JK (2021). Corona Concerts: The Effect of Virtual Concert Characteristics on Social Connection and Kama Muta. Front Psychol.

[215] Nordoff P, Robbin C (1971). Therapy in music for handicapped children.

[216] Geretsegger M, Fusar-Poli L, Elefant C, Mössler KA, Vitale G, Gold C. Music Therapy for Autistic People. Cochrane Database Syst. Rev. 2022;CD004381. 10.1002/14651858.CD004381.pub4. 10.1002/14651858.CD004381.pub4 PMC9082683 35532041

[217] Alvin J, Warwick A (1991). Music therapy for the autistic child.

[218] Berger DS (2002). Music therapy, sensory integration, and the autistic child.

[219] Whipple J (2004). Music in intervention for children and adolescents with autism: a meta-analysis. J Music Ther.

[220] Thompson GA, Mcferran KS, Gold C (2014). Family-centred music therapy to promote social engagement in young children with severe autism spectrum disorder: a randomized controlled study. Child Care Health Dev.

[221] Mayer-Benarous H, Benarous X, Vonthron F, Cohen D (2021). Music therapy for children with autistic spectrum disorder and/or other neurodevelopmental disorders: a systematic review. Front Psychiatry.

[222] Yu Q, Li E, Li L, Liang W (2020). Efficacy of interventions based on applied behavior analysis for autism spectrum disorder: a meta-analysis. Psychiatry Investig.

[223] Gold C, Solli HP, Krüger V, Lie SA (2009). Dose-response relationship in music therapy for people with serious mental disorders: systematic review and meta-analysis. Clin Psychol Rev.

[224] Geretsegger M, Mössler KA, Bieleninik L, Chen XJ, Heldal TO, Gold C (2017). Music therapy for people with schizophrenia and schizophrenia-like disorders. Cochrane Database Syst Rev.

[225] Leucht S, Leucht C, Huhn M, Chaimani A, Mavridis D, Helfer B (2017). Sixty years of placebo-controlled antipsychotic drug trials in acute schizophrenia: Systematic review, Bayesian meta-analysis, and meta-regression of efficacy predictors. Am J Psychiatry.

[226] Brotons M, Koger SM (2000). The impact of music therapy on language functioning in dementia. J Music Ther.

[227] Lord TR, Garner JE (1993). Effects of music on Alzheimer patients. Percept Mot Skills.

[228] Hanser SB (2021). The effectiveness of music-based interventions for dementia: An umbrella review. Music Med.

[229] Tsoi KKF, Chan JYC, Ng YM, Lee MMY, Kwok TCY, Wong SYS (2018). Receptive music therapy is more effective than interactive music therapy to relieve behavioral and psychological symptoms of dementia: a systematic review and meta-analysis. J Am Med Dir Assoc.

[230] Good A, Kreutz G, Choma B, Fiocco A, Russo F (2020). The SingWell project protocol: the road to understanding the benefits of group singing in older adults. Public Heal Panor.

[231] McDonald C, Stewart L (2008). Uses and functions of music in congenital amusia. Music Percept.

[232] Gladwell M, Headlam B (2021). Miracle and wonder: conversations with Paul Simon [Audiobook].

[233] Peretz I, Champod AS, Hyde K (2003). Varieties of musical disorders. Ann N. Y Acad Sci.

[234] Müllensiefen D, Gingras B, Musil J, Stewart L (2014). The musicality of non-musicians: an index for assessing musical sophistication in the general population. PLoS One.

[235] Hannon EE, Trainor LJ (2007). Music acquisition: effects of enculturation and formal training on development. Trends Cogn Sci.

[236] McDermott JH, Schultz AF, Undurraga EA, Godoy RA (2016). Indifference to dissonance in native Amazonians reveals cultural variation in music perception. Nature.

[237] Smit EA, Milne AJ, Sarvasy HS, Dean RT (2022). Emotional responses in Papua New Guinea show negligible evidence for a universal effect of major versus minor music. PLoS One.

[238] Drayna D, Manichaikul A, De Lange M, Snieder H, Spector T (2001). Genetic correlates of musical pitch recognition in humans. Science.

[239] Mosing MA, Madison G, Pedersen NL, Kuja-Halkola R, Ullén F (2014). Practice does not make perfect: no causal effect of music practice on music ability. Psychol Sci.

[240] Seesjärvi E, Särkämö T, Vuoksimaa E, Tervaniemi M, Peretz I, Kaprio J (2016). The nature and nurture of melody: a twin study of musical pitch and rhythm perception. Behav Genet.

[241] Pulli K, Karma K, Norio R, Sistonen P, Göring HHH, Järvelä I (2008). Genome-wide linkage scan for loci of musical aptitude in Finnish families: Evidence for a major locus at 4q22. J Med Genet.

[242] Oikkonen J, Huang Y, Onkamo P, Ukkolva-Vuoti L, Raijas P, Karma K (2015). A genome-wide linkage and association study of musical aptitude identifies loci containing genes related to inner ear development and neurocognitive functions. Mol Psychiatry.

[243] Niarchou M, Gustavson J, Sathirapongsasuti JF, Angelda-Tort M, Eising E, Bell E (2022). Genome-wide association study of musical beat synchronization demonstrates high polygenicity. Nat Hum Behav.

[244] Song Z, Albers HE (2018). Cross-talk among oxytocin and arginine-vasopressin receptors: relevance for basic and clinical studies of the brain and periphery. Front Neuroendocrinol.

[245] Ukkola LT, Onkamo P, Raijas P, Karma K, Järvelä I (2009). Musical aptitude Is associated with AVPR1A-haplotypes. PLoS One.

[246] Granot RY, Frankel Y, Gritsenko V, Lerer E, Gritsenko I, Bachner-Melman R (2007). Provisional evidence that the arginine vasopressin 1a receptor gene is associated with musical memory. Evol Hum Behav.

[247] Ukkola-Vuoti L, Oikkonen J, Onkamo P, Karma K, Raijas P, Järvelä I (2011). Association of the arginine vasopressin receptor 1A (AVPR1A) haplotypes with listening to music. J Hum Genet.

[248] Bachner-Melman R, Dina C, Zohar AH, Constantini N, Lerer E, Hoch S (2005). AVPR1a and SLC6A4 gene polymorphisms are associated with creative dance performance. PLoS Genet.

[249] Zhang Y, Zhu D, Zeng P, Le W, Qin W, Liu F (2020). Neural mechanisms of AVPR1A RS3-RS1 haplotypes that impact verbal learning and memory. Neuroimage.

[250] Levin R, Heresco-Levy U, Bachner-Melman R, Israel S, Shalev I, Epstien RP (2009). Association between arginine vasopressin 1a receptor (AVPR1a) promoter region polymorphisms and prepulse inhibition. Psychoneuroendocrinology.

[251] Meyer-Lindenberg A, Kolachana B, Gold B, Olsh A, Nikodemus KK, Mattay V (2009). Genetic variants in AVPR1A linked to autism predict amygdala activation and personality traits in healthy humans. Mol Psychiatry.

[252] Knafo A, Israel S, Darvasi A, Bachner-Melman R, Uzefovsky F, Cohen L (2008). Individual differences in allocation of funds in the dictator game associated with length of the arginine vasopressin 1a receptor RS3 promoter region and correlation between RS3 length and hippocampal mRNA. Genes, Brain Behav.

[253] Walum H, Westberg L, Henningsson S, Neiderhiser JM, Reiss D, Igl W (2008). Genetic variation in the vasopressin receptor 1a gene (AVPR1A) associates with pair-bonding behavior in humans. Proc Natl Acad Sci USA.

[254] Francis SM, Kim SJ, Kistner-Griffin E, Guter S, Cook EH, Jacob S (2016). ASD and genetic associations with receptors for oxytocin and vasopressin-AVPR1A, AVPR1B, and OXTR. Front Neurosci.

[255] Morley AP, Narayanan M, Mines R, Molokhia A, Baxter S, Craig G (2012). AVPR1A and SLC6A4 polymorphisms in choral singers and non-musicians: a gene association study. PLoS One.

[256] Ayotte J, Peretz I, Hyde K (2002). Congenital amusia: a group study of adults afflicted with a music-specific disorder. Brain.

[257] Grant Allen I (1878). Note deafness. Mind.

[258] Phillips-Silver J, Toiviainen P, Gosselin N, Piché O, Nozaradan S, Palmer C (2011). Born to dance but beat deaf: a new form of congenital amusia. Neuropsychologia.

[259] Mas-Herrero E, Zatorre RJ, Rodriguez-Fornells A, Marco-Pallarés J (2014). Dissociation between musical and monetary reward responses in specific musical anhedonia. Curr Biol.

[260] Peretz I, Cummings S, Dubé M-P (2007). The genetics of congenital amusia (tone deafness): a family-aggregation study. Am J Hum Genet.

[261] Loui P, Alsop D, Schlaug G (2009). Tone deafness: a new disconnection syndrome?. J Neurosci.

[262] Hyde KL, Zatorre RJ, Peretz I (2011). Functional MRI evidence of an abnormal neural network for pitch processing in congenital amusia. Cereb Cortex.

[263] Peretz I (2016). Neurobiology of congenital amusia. Trends Cogn Sci.

[264] Peretz I, Vuvan DT (2017). Prevalence of congenital amusia. Eur J Hum Genet.

[265] Mas-Herrero E, Maarco-Pallares J, Lorenzo-Seva U, Zatorre RJ, Rodriguez-Fornells A (2013). Individual differences in music reward experiences. Music Percept.

[266] Edwards J, Edwards J (2016). Conceptualizing music therapy: five areas that frame the field. The Oxford handbook of music therapy.

[267] Rolvsjord R, Edwards J (2016). Resource-oriented perspectives in music therapy. The Oxford handbook of music therapy.

[268] Vuvan DT, Paquette S, Mignault Goulet G, Royal I, Felezeu M, Peretz I (2018). The Montreal protocol for identification of amusia. Behav Res Methods.

[269] Peretz I, Gosselin N, Nan Y, Caron-Caplette E, Trehub SE, Beland R (2013). A novel tool for evaluating children’s musical abilities across age and culture. Front Syst Neurosci.

[270] Harrison PMC, Müllensiefen D (2018). Development and validation of the computerised adaptive beat alignment test (CA-BAT). Sci Rep.

[271] Constantino JN, Davis SA, Todd RD, Schindler MK, Gross MM, Brophy SL (2003). Validation of a brief quantitative measure of autistic traits: Comparison of the social responsiveness scale with the Autism Diagnostic Interview-Revised. J Autism Dev Disord.

[272] Liebowitz MR (1987). Social phobia. Mod Probl Pharmacopsychiatry.

[273] Bieleninik GeretseggerM, Mössler K, Assmus J, Thompson G, Gattino G (2017). Effects of improvisational music therapy vs enhanced standard care on symptom severity among children with autism spectrum disorder: the TIME-A randomized clinical trial. JAMA - J Am Med Assoc.

[274] Sharda M, Tuerk C, Chowdhury R, Jamey K, Foster N, Custo-Blanch M (2018). Music improves social communication and auditory–motor connectivity in children with autism. Transl Psychiatry.

[275] Greenberg DM, Kosinski M, Stillwell DJ, Montiero BL, Levitin D, Rentfrow PJ (2016). The song is you: preferences for musical attribute dimensions reflect personality. Soc Psychol Personal. Sci.

[276] Schedl M (2019). Deep learning in music recommendation systems. Front Appl Math Stat.

[277] Pereira CS, Teixeira J, Figueiredo P, Xaiver J, Castro SL, Brattico E (2011). Music and emotions in the brain: familiarity matters. PLoS One.

[278] van den Bosch I, Salimpoor VN, Zatorre RJ (2013). Familiarity mediates the relationship between emotional arousal and pleasure during music listening. Front Hum Neurosci.

[279] Freitas C, Manzato E, Burini A, Taylor MJ, Lerch JP, Anagnostou E (2018). Neural correlates of familiarity in music listening: a systematic review and a neuroimaging meta-analysis. Front Neurosci.

[280] Silverman MJ, Letwin L, Nuehring L (2016). Patient preferred live music with adult medical patients: a systematic review to determine implications for clinical practice and future research. Arts Psychother.

[281] Hilliard RE (2004). A post-hoc analysis of music therapy services for residents in nursing homes receiving hospice care. J Music Ther.

[282] Jiang J, Rickson D, Jiang C (2016). The mechanism of music for reducing psychological stress: music preference as a mediator. Arts Psychother.

[283] Sakka LS, Saarikallio S (2020). Spontaneous music-evoked autobiographical memories in individuals experiencing depression. Music Sci.

[284] Garrido S, Schubert E (2015). Moody melodies: do they cheer us up? A study of the effect of sad music on mood. Psychol Music.

[285] Yoon S, Verona E, Schlauch R, Schneider S, Rottenberg J (2019). Why do depressed people prefer sad music?. Emotion.

[286] Millgram Y, Joormann J, Huppert JD, Tamir M (2015). Sad as a matter of choice? Emotion-regulation goals in depression. Psychol Sci.

[287] Huron D, Vuoskoski JK (2020). On the enjoyment of sad music: pleasurable compassion theory and the role of trait empathy. Front Psychol.

[288] Sachs ME, Damasio A, Habibi A (2015). The pleasures of sad music: a systematic review. Front Hum Neurosci.

[289] Kenny DT, Asher A (2016). Life expectancy and cause of death in popular musicians: is the popular musician lifestyle the road to ruin?. Med Probl Perform Art.

[290] Anderson CA, Berkowitz L, Donnerstein E, Huessmann LR, Johnson JD, Linz D (2003). The influence of media violence on youth. Psychol Sci Public Interes.

[291] Thompson WF, Geeves AM, Olsen KN (2019). Who enjoys listening to violent music and why?. Psychol Pop Media Cult.

[292] Depasquale J. A fly over the fringes: depressive suicide black metal, past, present, and beyond. Invisible Oranges. 2018. https://www.invisibleoranges.com/depressive-black-metal/.

[293] Arroyo-Anlló EM, Díaz JP, Gil R (2013). Familiar music as an enhancer of self-consciousness in patients with Alzheimer’s disease. Biomed Res Int.

[294] Smith TW (1994). Generational differences in musical preferences. Pop Music Soc.

[295] Omigie D, Müllensiefen D, Stewart L (2012). The experience of music in congenital amusia. Music Percept.

[296] Fink LK, Warrenburg L, Howlin C, Randall WM, Hansen NC, Wald-Fuhrmann M (2021). Viral tunes: changes in musical behaviours and interest in coronamusic predict socio-emotional coping during COVID-19 lockdown. Humanit Soc. Sci Commun.

[297] North AC, Hargreaves DJ, O’Neill SA (2000). The importance of music to adolescents. Br J Educ Psychol.

[298] Tarrant M, North AC, Hargreaves DJEnglish (2000). and American adolescents’ reasons for listening to music. Psychol Music.

[299] Boer D, Fischer R, Tekman HG, Abubakar A, Njenga J, Zenger M (2012). Young people’s topography of musical functions: personal, social and cultural experiences with music across genders and six societies. Int J Psychol.

[300] West M, Horden P (2000). Music Therapy in Antiquity. Music as medicine: the history of music therapy since antiquity.

[301] Singh M. The cultural evolution of shamanism. Behav. Brain Sci. 2017;1–83. 10.1017/S0140525X17001893 28679454

[302] Golden TL, Tetreault L, Ray CE, Kuge MN, Tiedemann A, Magsamen S (2022). The state of music-based interventions for mental illness: thought leaders on barriers, opportunities, and the value of interdisciplinarity. Community Ment Health J.

[303] Gaston ET (1945). Music education for health. Music Educ J.

[304] Ross A. Classical view; Listening to prozac…Er, Mozart. *The New York Times*. August 28, 1994, Section 2, Page 23.

[305] Pietschnig J, Voracek M, Formann AK (2010). Mozart effect-shmozart effect: a meta-analysis. Intelligence.

[306] Nantais KM, Schellenberg EG (1999). The mozart effect: an artifact of preference. Psychol Sci.

[307] Kirsch I (2005). Placebo psychotherapy: synonym or oxymoron?. J Clin Psychol.

[308] Rolvsjord R, Gold C, Stige B (2005). Research rigour and therapeutic flexibility: Rationale for a therapy manual developed for a randomised controlled trial. Nord J Music Ther.

[309] Bradt J (2012). Randomized controlled trials in music therapy: guidelines for design and implementation. J Music Ther.

[310] Cuijpers P, Karyotaki E, Reijnders M, Ebert DD (2019). Was Eysenck right after all? A reassessment of the effects of psychotherapy for adult depression. Epidemiol Psychiatr Sci.

[311] Kim YK, Kim SM, Myoung H (2011). Musical intervention reduces patients’ anxiety in surgical extraction of an impacted mandibular third molar. J Oral Maxillofac Surg.

[312] Bringman H, Giesecke K, Thörne A, Bringman S (2009). Relaxing music as pre-medication before surgery: a randomised controlled trial. Acta Anaesthesiol Scand.

[313] Ribeiro MKA, Alcântara-Silva TRM, Oliverira JCM, Paula TC, Dutra JBR, Pedrino GR (2018). Music therapy intervention in cardiac autonomic modulation, anxiety, and depression in mothers of preterms: randomized controlled trial. BMC Psychol.

[314] Erkkilä J, Punkanen M, Fachner J, Ala-Ruona E, Pöntiö I, Tervaniemi M (2011). Individual music therapy for depression: randomised controlled trial. Br J Psychiatry.

[315] Rabeyron T, Robledo del Canto JP, Carasco E, Bisson V, Bodeau M, Vrait FX (2020). A randomized controlled trial of 25 sessions comparing music therapy and music listening for children with autism spectrum disorder. Psychiatry Res.

[316] Volpe U, Gianoglio C, Autiero L, Marino ML, Facchini D, Mucci A (2018). Acute effects of music therapy in subjects with psychosis during inpatient treatment. Psychiatry Interpers Biol. Process.

[317] Gold C, Mössler K, Grocke D, Heldal TO, Tjemsland L, Aarre T (2013). Individual music therapy for mental health care clients with low therapy motivation: multicentre randomised controlled trial. Psychother Psychosom.

[318] Gerdner LA (2000). Effects of individualized versus classical ‘relaxation’ music on the frequency of agitation in elderly persons with Alzheimer’s disease and related disorders. Int Psychogeriatr.

[319] Raglio A, Bellelli G, Traficante D, Gianotti M, Ubezio MC, Villani D (2008). Efficacy of music therapy in the treatment of behavioral and psychiatric symptoms of dementia. Alzheimer Dis Assoc Disord.

[320] Prince M, Patel V, Saxena S, Maj M, Maselko J, Phillips MR (2007). No health without mental health. Lancet.

[321] Czeisler MÉ, Wiley J, Facer-Childs ER, Robbins R, Weaver MD, Howard ME (2021). Mental health, substance use, and suicidal ideation during a prolonged COVID-19-related lockdown in a region with low SARS-CoV-2 prevalence. J Psychiatr Res.

[322] Moncrieff J, Kirsch I (2015). Empirically derived criteria cast doubt on the clinical significance of antidepressant-placebo differences. Contemp Clin Trials.

[323] Hengartner MP, Plöder M (2018). Statistically significant antidepressant-placebo differences on subjective symptom-rating scales do not prove that the drugs work: effect size and method bias matter!. Front Psychiatry.

[324] Leucht S, Kane J, Etschel E, Kissling W, Haman J, Engel RR (2006). Linking the PANSS, BPRS, and CGI: Clinical implications. Neuropsychopharmacology.

